# Reproductive constraints influence habitat accessibility, segregation, and preference of sympatric albatross species

**DOI:** 10.1186/s40462-015-0063-4

**Published:** 2015-09-29

**Authors:** Michelle A. Kappes, Scott A. Shaffer, Yann Tremblay, David G. Foley, Daniel M. Palacios, Steven J. Bograd, Daniel P. Costa

**Affiliations:** Department of Ecology and Evolutionary Biology, University of California Santa Cruz, 100 Shaffer Road, Santa Cruz, California 95060 USA; Environmental Research Division, Southwest Fisheries Science Center, NOAA Fisheries, 1352 Lighthouse Avenue, Pacific Grove, California 93950 USA; Joint Institute for Marine and Atmospheric Research, University of Hawai’i at Manoa, 1000 Pope Road, Honolulu, Hawai’i 96822 USA; Present address: Department of Fisheries and Wildlife, Oregon State University, 104 Nash Hall, Corvallis, Oregon 97331 USA; Present address: Department of Biological Sciences, San Jose State University, One Washington Square, San Jose, California 95192 USA; Present address: Institut pour la Recherche et le Développement, UMR 212 IRD-IFREMER-UM2. Av Jean Monnet 34200, Sète, France

**Keywords:** Laysan albatross, Black-footed albatross, Foraging behavior, Activity patterns, Spatial segregation, Habitat selection, Satellite tracking, Geolocation, Spatial modeling

## Abstract

**Background:**

The spatiotemporal distribution of animals is dependent on a suite of factors, including the distribution of resources, interactions within and between species, physiological limitations, and requirements for reproduction, dispersal, or migration. During breeding, reproductive constraints play a major role in the distribution and behavior of central place foragers, such as pelagic seabirds. We examined the foraging behavior and marine habitat selection of Laysan (*Phoebastria immutabilis*) and black-footed (*P. nigripes*) albatrosses throughout their eight month breeding cycle at Tern Island, Northwest Hawaiian Islands to evaluate how variable constraints of breeding influenced habitat availability and foraging decisions. We used satellite tracking and light-based geolocation to determine foraging locations of individuals, and applied a biologically realistic null usage model to generate control locations and model habitat preference under a case–control design. Remotely sensed oceanographic data were used to characterize albatross habitats in the North Pacific.

**Results:**

Individuals of both species ranged significantly farther and for longer durations during incubation and chick-rearing compared to the brooding period. Interspecific segregation of core foraging areas was observed during incubation and chick-rearing, but not during brooding. At-sea activity patterns were most similar between species during brooding; neither species altered foraging effort to compensate for presumed low prey availability and high energy demands during this stage. Habitat selection during long-ranging movements was most strongly associated with sea surface temperature for both species, with a preference for cooler ocean temperatures compared to overall availability. During brooding, lower explanatory power of habitat models was likely related to the narrow range of ocean temperatures available for selection.

**Conclusions:**

Laysan and black-footed albatrosses differ from other albatross species in that they breed in an oligotrophic marine environment. During incubation and chick-rearing, they travel to cooler, more productive waters, but are restricted to the low-productivity environment near the colony during brooding, when energy requirements are greatest. Compared to other albatross species, Laysan and black-footed albatrosses spend a greater proportion of time in flight when foraging, especially during the brooding period; this strategy may be adaptive for locating dispersed prey in an oligotrophic environment.

**Electronic supplementary material:**

The online version of this article (doi:10.1186/s40462-015-0063-4) contains supplementary material, which is available to authorized users.

## Background

To maximize fitness, animals can optimize energy acquisition through the selection of favorable habitats [1–4]. Foraging habitat use is constrained not only by the distribution of resources, but also by the physiological capabilities of the animal, memory and learned behaviors, intra- and interspecific interactions, and requirements for activities other than foraging, such as reproduction or migration [5–11].

Central place foragers, animals constrained by the need to return to a particular place (e.g., breeding colony) after a foraging trip, have a limited window of time, and therefore space, in which to search for and obtain food [12, 13]. Marine species that forage at sea while conducting breeding activities on land exemplify this behavior. The proximity of suitable foraging habitat to breeding sites has profound implications for behavior, habitat use, and energetics of central place foragers [14–20]. The extent to which marine animals are constrained in this context can depend on the particular stage of reproduction, especially when coupled with temporally changing energetic demands of offspring [15, 18, 21–24].

Albatrosses are pelagic seabirds that exhibit three distinct reproductive stages: (1) the incubation period (parents alternate between fasting at the nest to incubate the egg, and foraging at sea); (2) the brooding period (breeding pairs alternate between fasting at the nest to brood and provision the chick, and foraging at sea); and (3) the chick-rearing stage (breeding pairs forage independently at sea, returning to the nest periodically to quickly provision the chick). Differing energetic demands during these reproductive stages [18, 21, 25] constrain the duration and range of foraging movements, and ultimately influence the accessibility of foraging habitats to breeding adults [20, 26–29]. Habitat accessibility is additionally influenced by spatiotemporal variation in the distribution of preferred marine habitats e.g., [30–32].

Laysan (*Phoebastria immutabilis*) and black-footed (*P. nigripes*) albatrosses are long-ranging pelagic seabirds of the North Pacific that breed primarily in the warm, oligotrophic marine environment of the Northwest Hawaiian Islands [33, 34]. This is in contrast to other albatross species that nest in closer proximity to more productive marine habitats, such as subtropical and polar convergences, continental shelf-breaks and slopes, and coastal upwelling zones [35, 36]. Albatrosses use energetically efficient gliding flight to make long distance movements, allowing Laysan and black-footed albatrosses to travel to more productive habitats of the North Pacific during the incubation and chick-rearing stages [28, 29, 32]. During brooding, however, they are restricted to the oligotrophic waters close to their breeding colonies, where prey abundance is likely lower [37, 38]. Even the equatorially nesting waved albatross (*Phoebastria irrorata*) has access to higher productivity waters during brooding due to the close proximity of the Humboldt Current, equatorial fronts, and localized upwelling [39].

According to a model developed by Ricklefs [21], pelagic seabirds expend more energy per day during brooding than incubation or chick-rearing. This is due to the fact that brooding adults fast while providing the daily needs of the rapidly-growing chick, whereas chick-rearing adults provide half of the chick’s daily energy requirements and do not need to fast [40]. In the wandering albatross (*Diomedea exulans*), individuals respond by expending more energy while foraging during brooding compared to incubation [18], which allows them to maximize the rate of energy delivery to chicks during this time-limited breeding stage [18, 41]. It has been suggested that some albatrosses may couple the high energetic demands of the brooding period with seasonal increases in prey abundance [15], however, it is unknown how Hawaiian albatrosses locate sufficient prey resources to meet these high energetic demands when foraging in a low-productivity environment. Other top predators that make use of oligotrophic environments may exhibit lower abundances in the least productive waters or use low-productivity waters primarily for breeding, rather than feeding, activities [42–44].

Both Laysan and black-footed albatrosses are known to extensively use an oceanic region known as the North Pacific Transition Zone (NPTZ) when making long-ranging movements [28, 29, 32]. The NPTZ is bounded by the eastward flowing currents of the subtropical and subarctic gyres [45], and exhibits a sharp meridional transition in surface phytoplankton chlorophyll-*a* concentration, a feature known as the Transition Zone Chlorophyll Front [TZCF; [46]. The dynamics of the front act to aggregate phytoplankton and particulate matter, attracting mobile organisms; because zooplankton and other actively swimming or buoyant organisms can maintain their position in the front, the resulting prey aggregation serves to attract higher-trophic-level predators [46–49]. The latitudinal positional of the TZCF fluctuates on seasonal, interannual, and decadal time scales [50]; this spatiotemporal variation alters the distribution of marine habitats used by Hawaiian albatrosses and their proximity to breeding colonies. Expansion of oligotrophic habitat in the subtropical gyre of the North Pacific [51], as well as changes to the positioning of the North Pacific Current [52] due to climate change, could have negative effects on Laysan and black-footed albatross populations if preferred habitats become more distant from the colony during critical portions of the breeding season.

Here we examine the foraging behavior and habitat preference of sympatrically nesting Hawaiian albatrosses throughout the breeding cycle in order to evaluate how reproductive constraints impact foraging distribution, habitat segregation, at-sea activity patterns, and habitat preference in the context of their oligotrophic breeding environment. Based on previous studies, we expected that foraging movements would be most constrained during the brooding period for both species and least constrained during the incubation and chick-rearing periods [28, 29, 32]. We also expected to see the greatest overlap in foraging distributions during brooding when adults take shorter trips to frequently provision young chicks [29]. We therefore hypothesized that activity patterns of Hawaiian albatrosses during brooding would differ from the incubation and chick-rearing periods in response to (1) the need to maximize energy delivery to rapidly-growing chicks, (2) the use of a low-productivity foraging environment, and (3) the greater potential for inter- and intraspecific competition due to contraction of foraging ranges. We also hypothesized that greater interspecific differences in activity patterns would be observed during brooding; divergent foraging strategies could reduce competitive interactions between species despite high overlap in foraging distributions. Finally, we hypothesized that each species would display consistent environmental associations in selecting habitats throughout the breeding season, but that reproductive constraints and seasonal variation in marine habitats would influence the composition of utilized habitats during the different breeding stages.

## Methods

### Study area and tracking activities

Breeding Laysan and black-footed albatrosses were studied at Tern Island (23.87° N, 166.28° W), French Frigate Shoals, Northwest Hawaiian Islands, during the incubation, brooding, and chick-rearing periods. The incubation period for both species lasts approximately 60 days, beginning in mid-late November with the laying of a single egg and ending with the onset of hatching in late January. The brooding period typically lasts 2–3 weeks, until chicks are left alone at the nest in mid-February. The chick-rearing period lasts approximately 4–5 months; after a period of fasting, chicks fledge independently in June and July [33, 34].

We used a combination of satellite tracking and light-level based geolocation to determine at-sea locations of adult Hawaiian albatrosses throughout the reproductive period. Satellite tags were used for short-term deployments during incubation and brooding, whereas geolocation tags were used to obtain foraging positions during chick-rearing, and to supplement data from the incubation period. While geolocation tag deployments also spanned the brooding period, the single position derived per day by light-level based geolocation did not have sufficient resolution to accurately capture movements during brooding when average trip durations were 2–3 days (Table 1; satellite tags are accurate to <10 km [53]; geolocation tags are accurate to ~200 km [54]). Tracking activities were conducted during five consecutive breeding seasons, from 2002–03 through 2006–07; satellite tracks were obtained during the 2002–03 to 2005–06 seasons and geolocation data were obtained during the 2003–04 to 2006–07 seasons. Sex of tracked individuals was determined from blood samples [55]; for six individuals for which we did not obtain blood samples, sex was determined by comparison of culmen lengths [28].

**Table 1:** Summary characteristics (Mean ± SD) of Laysan and black-footed albatross foraging trips. To reduce the influence of individuals tracked for multiple foraging trips, a single trip per individual was randomly-selected for each reproductive stage to include in the calculation of mean values

Species		Incubation	Brooding	Chick-Rearing
Laysan albatross	Number of individuals tracked	58	38	26
	Total number of foraging trips	72	40	153
	Trip duration (days)	17.6 ± 7.45 ^a,*^	2.62 ± 0.76^b^	14.5 ± 3.91^c^
	Maximum distance from colony (km)	2433 ± 837^a^	420 ± 266^b^	2489 ± 676^a,*^
	Azimuth to most distant point from colony (°)	350 ± 26.5^a,*^	35.4 ± 64.5^b^	358 ± 32.4^a,*^
Black-footed albatross	Number of individuals tracked	51	35	23
	Total number of foraging trips	60	37	83
	Trip duration (days)	13.3 ± 5.07^a,*^	2.67 ± 0.87^b^	14.4 ± 5.14^a^
	Maximum distance from colony (km)	2045 ± 1035^a^	313 ± 144^b^	2883 ± 998^c,*^
	Azimuth to most distant point from colony (°)	22.8 ± 32.5^*^	47.4 ± 65.7	31.9 ± 35^*^

Different lowercase letters indicate significant differences (*P* < 0.05) among reproductive stages; asterisks indicate significant differences between species

### Satellite tracking

One hundred and forty-seven adult albatrosses (76 Laysan and 71 black-footed) were equipped with satellite platform terminal transmitters (30 g Pico-100, Microwave Telemetry, Columbia, MD; or 35 g SPOT4, Wildlife Computers, Redmond, WA); tags were attached to dorsal feathers with adhesive tape (tesa®, Hamburg, Germany), and transmissions were downloaded via the Argos satellite system (Service Argos, Inc., Largo, MD). Satellite tags were programmed to transmit continuously every 90 s, with the exception of 19 foraging trips, when tags were programmed to use a 6:18 h on:off duty cycle (11 trips), 20:4 h on:off duty cycle (6 trips), or 9:15 h on:off duty cycle (2 trips) to conserve battery life on anticipated longer trips. In three instances, the battery on the satellite transmitter failed or the satellite transmitter fell off the bird before completion of the foraging trip. During 2002–03, 2004–05, and 2005–06, satellite-tracked individuals were also equipped with archival tags (10 g Lotek LTD 2400, Lotek Wireless, St. John’s, Newfoundland) attached to a plastic leg band so that temperature recordings (±0.05 °C) every 12–40 s could be used to characterize foraging activity while at sea [56]. The combined mass of devices deployed on individuals was less than 2 % of total bird body mass in all cases, below the recommended limit for studies involving albatrosses [57].

Before calculating trip characteristics, satellite locations were first delimited by observations of departure and arrival times at the breeding colony or by visual inspection of the tracks. To remove unlikely locations from the data set, we applied an iterative forward/backward averaging speed filter [58] implemented in Matlab (The MathWorks, Natick, MA) with a maximum speed limit of 80 km h^−1^ (following [29, 59]) to remove unrealistic flight speeds [60]. Satellite tracks were then interpolated to every 10 min using a Bézier curve with μ = 0.3 [μ controls the elasticity of the curve; following [61], and subsampled to two locations per day to match the temporal scale of geolocation positions (see below).

### Tracking with geolocation tags

Archival geolocation tags (10 g Lotek LTD 2400, Lotek Wireless, St. John’s, Newfoundland) were deployed and successfully recovered from 34 Laysan and 26 black-footed albatrosses. Each tag recorded ambient light intensity and temperature every 480 s or 540 s to determine a single daily location: longitude based on the establishment of local noon in comparison to Universal Time, and latitude based on day length for the established longitude [62, 63]. Temperature sensors on the geolocation tags allowed a refinement of the location data based on sea surface temperature [SST; [54, 64], as well as providing a record of foraging activity while at sea.

Geolocation tags were deployed for up to one year, only a portion of which overlapped our on-colony research activities, therefore we had limited information on the presence or absence of geolocation-tagged individuals at the colony. Due to the nature of the SST-processing algorithm [54, 64], it was necessary to first delimit individual foraging trips so that on-colony locations were removed from the dataset. We calculated minimum daily temperatures recorded by geolocation tags to determine days when albatrosses were likely at sea; evaporative cooling due to immersion in water would lead to cooler minimum temperatures on days when albatrosses were off-colony. In order to detect shifts in minimum daily temperatures indicative of on- or off-colony periods, we used an algorithm developed for detecting climate regime shifts [65], which implements a sequential version of the partial CUSUM method combined with the t-test, and is available for download at: http://www.beringclimate.noaa.gov/regimes/. For those individuals for which exact colony attendance patterns were known (25 individuals during incubation), this detection algorithm correctly classified 99.0 % of days when birds were known to be on-colony and 83.6 % of days when birds were known to be at-sea (*n* = 548 bird days). This method was therefore effective in delimiting foraging departures, but some at-sea locations may have been excluded. Estimated departure and arrival times for geolocation-derived foraging trips were calculated based on Argos-derived transit rates during the incubation period [32], and distance to the colony from the first and last off-colony location.

To remove unlikely light-based positions, we applied the same speed filter as above, with a more conservative speed limit of 50 km h^−1^ to account for greater error in geolocation position estimates [54], and interpolated to two positions per day using a Hermite spline [following [61].

### Foraging movements

Descriptive characteristics of all Argos- and geolocation-derived tracks were calculated to compare foraging behavior during each reproductive stage. Maximum distance traveled from the colony was calculated using great-circle distances to account for the earth’s curvature. Destination bearings, as defined by the azimuth to the most distant point, were calculated to describe overall direction of foraging trips. Trip duration was defined as either the time elapsed between the observed departure and arrival of the bird, or the time elapsed between the estimated departure and arrival times.

We used linear mixed-effects models [66], with individual as a random effect, to compare maximum ranges and trip durations between species and reproductive stages. Mixed-effects models were followed by contrast analysis with the multcomp package [67] in the program R [68]. To compare destination bearings between species and breeding stages, we used Watson-Williams tests for circular data in the CircStat toolbox in Matlab [69] after ensuring data followed the von Mises distribution (equivalent to normal distribution for circular data). Accounting for random effects has not been developed in the circular statistics modeling framework, therefore we randomly selected a single trip for each individual during each reproductive stage for this analysis.

### Foraging distribution

To determine patterns of interspecific habitat segregation during each reproductive stage, we used kernel estimation [70] to determine utilization distributions (UD) for each species. Because individuals contributed varying numbers of foraging trips to the overall data set, a single trip was randomly selected for each individual during each reproductive stage so that the influence of individual behaviors on estimation of kernel densities was reduced. Geographic coordinates of interpolated albatross locations were transformed using a Lambert Cylindrical Equal Area projection [71], and UDs were computed on a 50-km grid using the R package adehabitat [72]. To allow comparisons between species, the smoothing factor (h) was set to the mean of the h values calculated from each species, as determined using least-squares cross-validation [73]. We then employed a randomization analysis to test the null hypotheses that there was no spatial segregation in foraging distributions between species during each reproductive stage [74]. For each comparison, species was randomly assigned to tracks using the same species ratio observed, and kernel analysis applied. The area of overlap divided by the area of the larger of the two UD polygons was used as the test statistic following Breed et al. [74], for the 25 % (core area), 50 % (focal region), and 95 % (foraging range) UDs [29]. Each test was iterated 500 times, and the *P*-value was calculated as the proportion of random overlaps smaller than the observed overlap [74].

### Activity patterns at sea

We determined the proportion of time albatrosses spent in flight and the frequency of landings on the sea surface during daylight and nighttime hours to characterize foraging activity patterns during each reproductive stage using geolocation tags equipped with temperature sensors. Albatrosses are surface-feeders and, by necessity, must land on the sea surface in order to consume prey. Therefore, landing rates are indicative of the level of feeding effort. Flight costs measured for albatrosses have demonstrated that the most energetically demanding activities albatrosses engage in at sea are take-offs and landings [75]; landing rates were also highly correlated with field metabolic rates in a study of wandering albatrosses [76].

For albatrosses equipped with temperature recorders, we implemented an algorithm (Iknos toolkit for Matlab; Y. Tremblay, unpublished) designed to identify landings based on rapid changes in temperature, and stable periods associated with sitting on the sea surface [56]. We used civil twilight (sun no more than 6° below the horizon) to define daylight hours, based on temporally-matched tracking locations and NOAA’s solar calculator, as implemented in the maptools package in R [77] .

To test whether percent time in flight and landing rates differed between high-resolution temperature records (12–40 s) and low-resolution records (480 s and 540 s), we subsampled high resolution records to the lower resolution and compared these metrics. While percent time in flight did not differ between high resolution and subsampled records (paired t-test: *t*_140_ = 1.11, *P* = 0.27), landing rates were significantly lower in the subsampled group (paired t-test: *t*_140_ = 20.9, *P* < 0.0001). To compare all temperature records in subsequent analyses (only low resolution records were available during the chick-rearing period), we used histogram matching to rescale landing rates from low resolution records [78]. Histogram matching is an image processing technique used to rescale lower resolution data so that the histogram of the data after transformation matches that of the reference data [79]. This allowed us to present landing rates comparable to values in the literature determined from high resolution temperature loggers; significance tests based on histogram-matched landing rates yielded the same conclusions as tests based on landing rates from all the data subsampled to the lowest resolution available.

Percent time in flight was arcsine transformed and landing rate was log transformed prior to analysis to meet normality assumptions. We used linear mixed-effects models [66] with individual as a random effect to compare percent time in flight and landing rates between species and reproductive stages, followed by contrast analysis with the multcomp package in R [67].

### Habitat preference

We followed the analytical framework of Aarts et al. [80] to model habitat preference of Laysan and black-footed albatrosses during each stage of breeding. We adopted a case–control design such that each tracking location was temporally matched with three randomly generated control locations. Because breeding albatrosses are central place foragers, it is unrealistic to assume that all points within the study area are equally accessible [80, 81]. Therefore, we adopted a simple null model of usage that assumes that the accessibility α [81] of a point in space is inversely related to the distance from the colony (d_c_) [82]. We assumed that locations were not accessible beyond the maximum range observed for each breeding stage for each species; control locations were then quasi-randomly selected at a rate proportional to α within this range for each species-stage combination. Because this null model may over- or under-predict true accessibility, we also included d_c_ as a candidate covariate in our habitat preference models [83] as suggested by Aarts et al. [80].

**Fig. 1:**
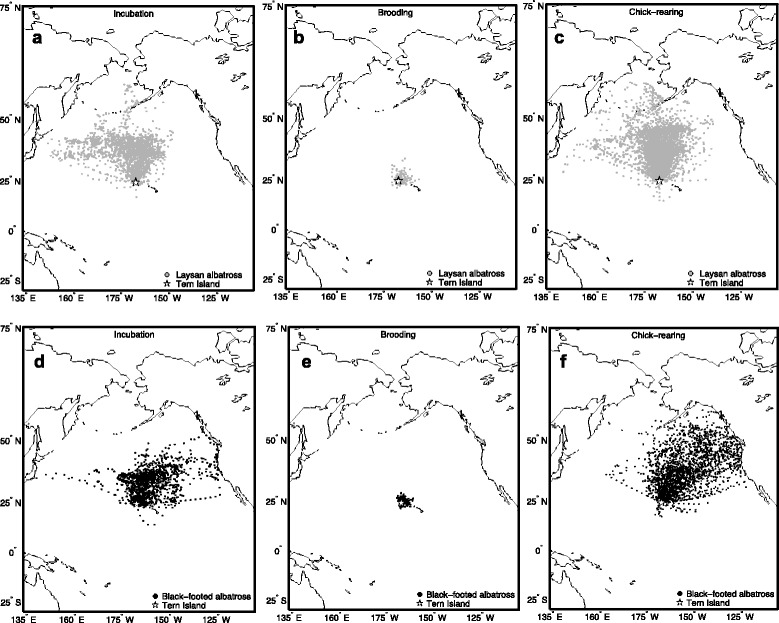
Tracking locations of Laysan and black-footed albatrosses breeding at Tern Island, Northwest Hawaiian Islands. Data encompass five breeding seasons, from 2002–03 through 2006–07, for Laysan albatrosses during incubation **a** brooding **b** and chick-rearing **c** and black-footed albatrosses during incubation **d** brooding **e** and chick-rearing **f**

Laysan and black-footed albatrosses breeding at Tern Island rarely made southward departures from the colony (Fig. 1); therefore we also modeled habitat preference using a more restrictive null model of usage to reflect the northern bias of tracking locations (see Appendix A in Additional file 1) and to ensure conclusions were not sensitive to the choice of null usage model. Final habitat models and response curves of selected covariates were generally similar irrespective of the null usage model (see Results and Appendix A in Additional file 1), therefore we present only the results based on the simpler null usage model, which assumed that locations were accessible within the maximum range observed for each species-stage, regardless of the direction from the colony. This required fewer assumptions regarding accessibility of habitats, and followed the implementation of Wakefield et al. [82].

**Fig. 2:**
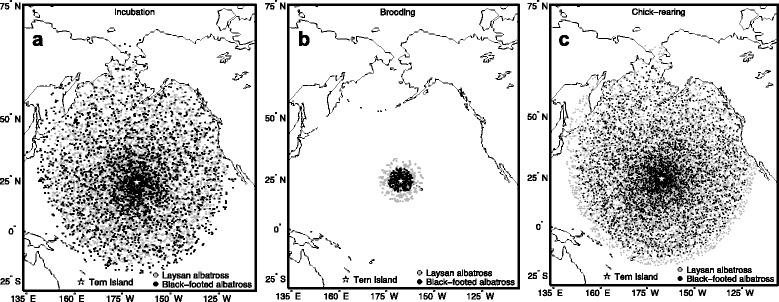
Randomly generated control locations selected at a rate proportional to accessibility**.** Maximum observed ranges of Laysan and black-footed albatrosses during the incubation **a** brooding **b** and chick-rearing **(c)** periods were used to limit spatial extent of control locations

Habitat preference models were implemented using a binomial distribution and logit link (the inverse of the logistic function) to relate the response (tracking locations, value of 1 (Fig. 1) and control locations, value of 0 (Fig. 2)) to environmental covariates. Geolocation tracks (incubation and chick-rearing) were only included in habitat analyses if they included at least five filtered off-colony locations, to ensure adequate coverage of sampled habitats along each track. We applied generalized additive mixed models (GAMMs) to allow for the possibility of a nonlinear response to environmental covariates [84], and to account for non-independence of points within trips and the variable number of trips contributed by each individual [85].

### Environmental covariates

We selected marine habitat variables based on their potential to characterize physical features that may stimulate or aggregate albatross prey resources. Because Hawaiian albatrosses are surface-feeders foraging within the top meter of the water column, they rely on neustonic or vertically migrating prey, as well as on carrion and fisheries discards [33, 34, 86, 87]. Hawaiian albatrosses consume a diverse array of prey items, with Ommastrephid squid and flying fish (Exocoetidae) eggs comprising the largest proportion of the Laysan and black-footed albatross diet, respectively [86]. The diversity of prey types consumed by Hawaiian albatrosses reflects the variety of marine habitats encountered during their long-ranging movements; for instance, flying fish eggs are likely unavailable to black-footed albatrosses when foraging in the temperate waters of the California Current, where squid may be a larger component of their diet.

Previous studies have demonstrated a relationship between catches of Ommastrephid squid and SST [88–90], productivity [91], and position relative to frontal features of the North Pacific Transition Zone (NPTZ) [90]. Areas of surface convergence, such as those found near fronts and eddies, can also aggregate algae or floating material which many species of flying fish use to attach their non-buoyant eggs with sticky filaments [92, 93]. Adult flying fish, also prey of Hawaiian albatrosses [86], can be found at the outer edges of rapidly rotating eddies [93, 94].

The habitat relationships of these prey items informed our selection of environmental parameters with which to investigate albatross habitat use. We used SST and primary productivity (PP) to characterize the regional thermal and phytoplankton production regimes; latitudinal gradients in SST and proximity to the Transition Zone Chlorophyll Front (TZCF) to describe large-scale frontal characteristics; wind stress curl to describe wind-driven oceanic upwelling or downwelling; and sea surface height anomaly (SSHa) and eddy kinetic energy (EKE) to investigate overall intensity of eddy activity [95–97]. As albatrosses use near-surface winds to engage in gliding flight [98], we also characterized wind speed and direction for all locations used in the habitat analysis. Because Hawaiian albatrosses periodically visit the continental margins of the western coast of North America and the Aleutian Islands during breeding [28, 29, 32], and bathymetric features can stimulate local production and aggregate prey [99, 100], we also used sea floor depth to characterize marine habitats; bathymetry data was extracted from NOAA’s ETOPO2 data set (http://www.ngdc.noaa.gov/mgg/global/etopo2.html).

Environmental data were obtained by querying the NOAA OceanWatch Live Access Server using Matlab and ERDDAP (http://coastwatch.pfel.noaa.gov/erddap/). Where possible, we used satellite products with the finest temporal resolution available (daily or weekly); we used monthly composites when necessary to compensate for data gaps due to cloud cover.

We used a blended product of SST derived from both microwave and infrared sensors carried on multiple platforms at a spatial resolution of 0.1 degrees and as a 5-day temporal composite [101]. As SSTs in the central North Pacific are distributed in broad latitudinal bands, latitudinal gradients in SST (dySST) were used to describe frontal structure [102], by computing the local derivative of adjacent 0.1-degree pixels in the north–south direction.

Monthly PP estimates [103] were derived at a spatial resolution of 0.1 degrees from monthly chlorophyll-*a* values and photosynthetically available radiation obtained from the Sea-viewing Wide Field-of-view Sensor (SeaWiFS) on the Orbview-2 satellite, and SST values from the AVHRR Pathfinder Oceans Project.

Monthly chlorophyll-*a* values obtained from SeaWiFS were also used to calculate distance to the TZCF. Following Polovina et al. [46] and Bograd et al. [50], the TZCF was defined as the 0.2 mg m^−3^ chlorophyll-*a* contour.

Daily composites of wind velocity fields were gathered by the SeaWinds scatterometer aboard NASA’s QuikSCAT satellite at a resolution of 0.25 degrees and a reference height of 10 m, and used to assign daily values of wind speed and direction to each location.

The NOAA CoastWatch West Coast Node also processes wind vector fields to calculate wind stress curl from these data. We used wind stress curl as a metric of surface convergence/divergence [50], as a proxy for local aggregation of neustonic or buoyant prey (e.g., fish eggs, fish and squid larvae, zooplankton, fishing discards). For each location of interest, we calculated a mean wind stress curl from the previous 30 days to get a representation of past physical forcing in the area of interest.

EKE was calculated from surface ocean currents derived from four satellite altimeters (Jason-1, ENVISAT, ERS-1 and 2, and TOPEX/Poseidon) provided by the AVISO program at a resolution of 0.25 degrees. For each location of interest, a 10 x 10-degree spatial mean was removed from each 7-day composite of zonal and meridional surface ocean currents to generate zonal and meridional velocity anomalies (*u’* and *v’*). The EKE (per unit mass) was then calculated as

1 $$ EKE=\frac{1}{2}\left(u{\hbox{'}}^2+v{\hbox{'}}^2\right) $$

SSHa was derived from AVISO sea level data compared to the mean geoid as measured from 1993–1995.

Oceanographic data were extracted so that composite data matched closest in time to tag-derived locations (and matching control locations). The median value of each oceanographic variable was calculated for grid cells falling within the approximate error of Argos or geolocation positions. To estimate error of Argos locations, we conducted trials on seven satellite tags affixed to permanent structures at the University of California Santa Cruz, Long Marine Lab, for a period of three weeks. Argos locations were then compared to GPS-derived locations; mean error for all location qualities was 0.06° (6.7 km) from a total of 2,585 location fixes. To be conservative, satellite tag-derived locations (and matching control locations) were extracted within a 0.15° (16.7 km) longitude by 0.15° (16.7 km) latitude grid centered on each at-sea location. For geolocation-derived positions, data were extracted within a 1° (111 km) longitude by 2° (222 km) latitude grid centered on each location [the approximate error of the geolocation method; [54].

### Model fitting and selection

We modeled habitat preference for each species and breeding stage considering the following candidate covariates: SST (°C), PP (mg C m^-2.^day^−1^), dySST (°C km^−1^), minimum distance to the TZCF (dTZCF; km), wind stress curl (Pa m^−1^), SSHa (cm), EKE (cm^2^ s^−2^), wind speed (m s^−1^), wind direction (°), sea floor depth (m), and d_c_ (km). EKE and PP were log-transformed to achieve an even spread of covariate values, thus avoiding undue leverage of a few high values [104]. To ensure that there was not strong collinearity among parameters, we used generalized linear models with a binomial distribution and logit link to relate the response variable (presence/control) to all covariates, and calculated tolerance values (inverse of the variance inflation factor) for each species-stage; all tolerance values were greater than 0.1, the approximate guide suggested by Quinn and Keough [105].

GAMMs were implemented within the gamm4 package in R [106]. All candidate covariates were fitted using cubic regression splines; a cyclic spline was used to model wind direction. We first investigated the potential effects of transmitter duty-cycle and tag type (geolocation versus satellite tracking), as well as year and sex effects, by comparing intercept-only GAMMs for each species and reproductive stage to models including each of the above terms. In all cases, Akaike’s information criterion (AIC) values were not improved by the addition of these terms, therefore they were not subsequently included in models of habitat preference.

To arrive at an inferential model for each species and breeding stage, we used AIC as a guide, and relied on cluster-level cross-validation for final model selection [80]. This approach was taken due to the fact that model selection based on AIC alone can lead to overparameterized models when data are spatially or temporally autocorrelated, as is expected with both tracking and oceanographic data [80]. Therefore, we randomly selected two-thirds of the individuals tracked during each breeding stage for each species to use in the first stage of model selection. We started with intercept-only models and used forward model selection with AIC to arrive at a suite of candidate models for each species-stage. We then fit data from the remaining individuals to the sequence of models obtained from forward selection. The model with the lowest AIC from the cross-validation step was retained as the inferential model. To reduce the chance of over-fitting, we then replaced each spline in turn by a linear term and selected a final inferential model for each species-stage based on AIC values. Candidate GAMMs were fit using maximum likelihood to allow comparison of models with different fixed effect structures [107]; final inferential models were fit using restricted maximum likelihood estimation [108]. We used the proportion of the total deviance explained by the model to assess goodness of fit [107], and the percent deviance contributed by each coefficient to assess how much variability in the response could be explained by each main effect [109].

## Results

### Foraging movements

**Fig. 3:**
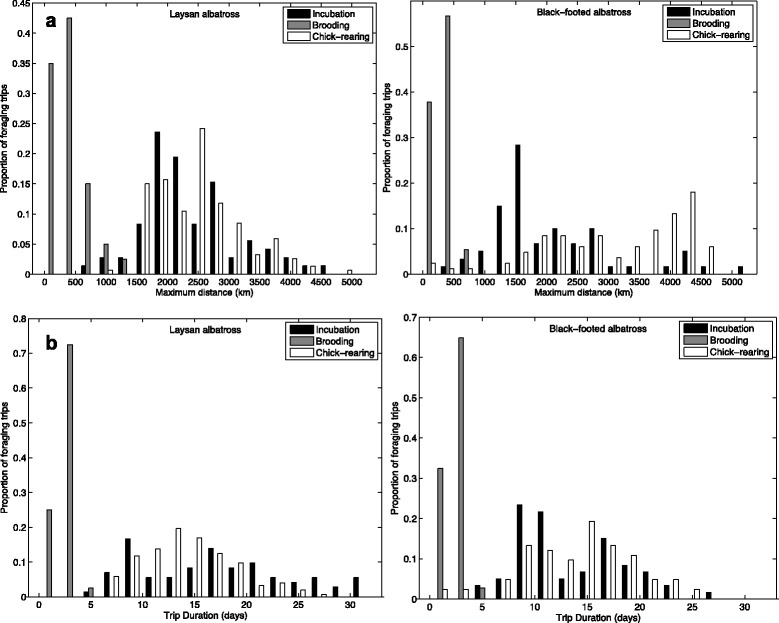
Distribution of foraging ranges (a) and trip durations (b) of Laysan and black-footed albatrosses

Both Laysan and black-footed albatrosses ranged significantly farther and were at sea for longer durations during incubation and chick-rearing, as compared to the brooding period (*P* < 0.001 for all pair-wise tests; Table 1). Although the majority of foraging trips occurred in pelagic waters of the North Pacific, Laysan and black-footed albatrosses regularly traveled to neritic environments of the Aleutian islands and the western coast of North America, respectively, especially during the chick-rearing period (Fig. 1). Black-footed albatrosses ranged farther during chick-rearing, as compared to the incubation period, while Laysan albatrosses had significantly longer trip durations during incubation as compared to chick-rearing (Table 1). In both species there was a greater spread in the distribution of trip lengths and foraging ranges during incubation and chick-rearing compared to brooding (Fig. 3a and b). Some overlap in foraging ranges and trip durations occurred during all three stages, when individuals took shorter trips during incubation and chick-rearing (Fig. 3a and b). Laysan albatrosses changed destination bearings between reproductive stages, whereas black-footed albatrosses demonstrated consistency in direction of travel between stages (Table 1).

Laysan albatrosses traveled for longer durations than black-footed albatrosses during the incubation period, however maximum distances did not differ between species during this stage (Table 1). Black-footed albatrosses traveled farther than Laysan albatrosses during chick-rearing, but species traveled similar distances during brooding. Azimuth to most distant point was significantly different between species during incubation and chick-rearing, with Laysan albatrosses demonstrating a more northerly distribution during incubation, and black-footed albatrosses demonstrating a more easterly distribution, especially during chick-rearing (Fig. 1; Table 1).

### Foraging distribution

**Fig. 4:**
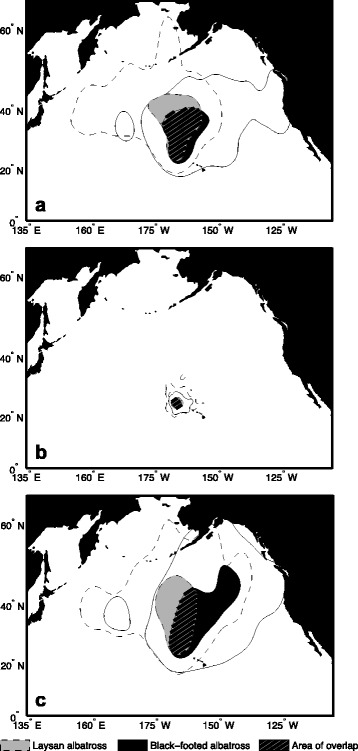
Overlap of Laysan and black-footed albatross focal foraging areas (50 % UD) and foraging ranges (95 % UD). Contours for the **a** incubation, **b** brooding, and **c** chick-rearing periods are outlined in dashed and solid lines for Laysan and black-footed albatrosses, respectively

Based on randomization tests of spatial overlap, Laysan and black-footed albatrosses demonstrated significant spatial segregation of focal (50 % UD) and core (25 % UD) foraging areas during incubation (focal: 64.7 % overlap, *P* = 0.014; core: 50.2 % overlap, *P* = 0.006) and chick-rearing (focal: 42.9 % overlap, *P* = 0.002; core: 24.8 % overlap, *P* = 0.004), but not during brooding (focal: 74.2 % overlap, *P* = 0.32; core: 72.0 % overlap, *P* = 0.35; Fig. 4). Overall foraging ranges (95 % UD) demonstrated a different pattern; during incubation as well as brooding, there was significant interspecific segregation of foraging ranges (incubation: 54.3 % overlap, *P* = 0.002; brooding: 51.0 % overlap, *P* = 0.012), but no spatial segregation of foraging ranges during chick-rearing (68.6 % overlap, *P* = 0.14; Fig. 4).

### Activity patterns at sea

Laysan albatrosses spent a greater proportion of time in flight during brooding (85.2 %), as compared to the incubation (74.0 %) and chick-rearing periods (78.5 %; Table 2). This difference was reflected in both the percent time spent in flight during the day and at night (Table 2). Black-footed albatrosses did not vary in terms of the proportion of time in flight between reproductive stages (82.6 % overall); however, landing rates were higher during chick-rearing (0.65 landings h^−1^) as compared to the incubation period (0.52 landings h^−1^; Table 2). This difference was accounted for by the greater frequency of landings during the day during chick-rearing (0.84 landings h^−1^) versus incubation (0.62 landings h^−1^); there was no significant difference in landing rate at night between reproductive stages (0.46 landings h^−1^ overall). Laysan albatrosses did not demonstrate differences in landing rate as a function of breeding stage (0.84 landings h^−1^ overall), either during the day (0.96 landings h^−1^ overall) or at night (0.72 landings h^−1^ overall).

**Table 2:** Summary characteristics (Mean ± SD) of at-sea activity patterns of Laysan and black-footed albatrosses. To reduce the influence of individuals tracked for multiple foraging trips, a single trip per individual was randomly-selected for each reproductive stage to include in the calculation of mean values

Species		Incubation	Brooding	Chick-Rearing
Laysan albatross	Time in flight (%)	74.0 ± 10.9 ^a,*^	85.2 ± 10.4 ^b^	78.5 ± 12.3 ^a^
	Day time in flight (%)	83.6 ± 8.68 ^a^	90.1 ± 7.6 ^b^	86.8 ± 9.08 ^a^
	Night time in flight (%)	66.0 ± 15.9 ^a,*^	80.4 ± 15.8 ^b^	66.8 ± 19.0 ^a^
	Landings per hour	0.85 ± 0.37 ^*^	0.85 ± 0.44	0.82 ± 0.44 ^*^
	Day landings per hour	0.93 ± 0.33 ^*^	0.98 ± 0.49	1.01 ± 0.49
	Night landings per hour	0.77 ± 0.49 ^*^	0.71 ± 0.51 ^*^	0.64 ± 0.45 ^*^
Black-footed albatross	Time in flight (%)	81.5 ± 8.26 ^*^	84.8 ± 10.2	81.3 ± 8.05
	Day time in flight (%)	84.9 ± 8.43	89.1 ± 7.72	86.9 ± 7.28
	Night time in flight (%)	78.6 ± 10.1 ^*^	80.5 ± 15.7	73.0 ± 12.2
	Landings per hour	0.52 ± 0.13 ^a,*^	0.71 ± 0.49 ^ab^	0.65 ± 0.17 ^b,*^
	Day landings per hour	0.62 ± 0.15 ^a,*^	0.92 ± 0.53 ^ab^	0.84 ± 0.25 ^b^
	Night landings per hour	0.41 ± 0.16 ^*^	0.52 ± 0.56 ^*^	0.46 ± 0.17 ^*^

Different lowercase letters indicate significant differences (*P* < 0.05) among reproductive stages; asterisks indicate significant differences between species

During incubation, black-footed albatrosses spent a greater proportion of time in flight compared to Laysan albatrosses (Table 2). Percent time in flight did not differ between species during daylight hours; however, black-footed albatrosses spent more time in flight at night during incubation compared to Laysan albatrosses (Table 2). Laysan albatrosses landed on the sea surface more frequently on average than black-footed albatrosses during incubation and chick-rearing. Laysan albatross landing rates during the day were more frequent than those of black-footed albatrosses during the incubation period only; landing rates at night were more frequent for Laysan albatrosses during all three breeding stages (Table 2).

Both species demonstrated diel patterns in foraging activity during all reproductive stages (Table 2). Laysan albatrosses spent a higher proportion of time in flight during the day than at night (incubation: *t*_100_ = 6.70, *P* < 0.0001; brooding: *t*_74_ = 3.51, *P* = 0.0008; chick-rearing: *t*_50_ = 5.10, *P* < 0.0001) and landed more frequently during the day (incubation: *t*_100_ = 3.08, *P* = 0.003; brooding: *t*_74_ = 2.97, *P* = 0.004; chick-rearing: *t*_50_ = 3.83, *P* = 0.0004). Black-footed albatrosses demonstrated the same pattern; they spent more time in flight during the day (incubation: *t*_86_ = 3.19, *P* = 0.002; brooding: *t*_68_ = 2.40, *P* = 0.02; chick-rearing: *t*_44_ = 4.43, *P* < 0.0001) and landed more frequently during daylight than at night (incubation: *t*_86_ = 6.51, *P* < 0.0001; brooding: *t*_68_ = 4.90, *P* = 0.0008; chick-rearing: *t*_44_ = 6.06, *P* < 0.0001).

### Habitat preference

Final inferential habitat preference models included environmental covariates descriptive of regional oceanic production (SST and PP), large-scale fronts (d_TZCF_), mesoscale activity (EKE and SSHa), and sea floor depth (Table 3). Environmental covariates not retained in final models were: dySST, wind stress curl, and wind speed or direction. SST was retained as a smooth term, and explained the greatest proportion of explained deviance in final habitat models selected for all reproductive stages for black-footed albatrosses, and during incubation and chick-rearing for Laysan albatrosses (46-76 %; Table 3).

**Table 3:** Model selection results from generalized additive mixed modeling of Laysan and black-footed albatross habitat preference. Final models were arrived at by forward selection based on AIC using a subset of individuals, followed by cross-validation using the remaining individuals

Laysan albatross	Model terms [% deviance explained by coefficient]						% deviance explained by model
Incubation	s(SST)	s(d_c_)	s(depth)	SSHa	s(d_TZCF_)	EKE	39.8 %
	[60.6]	[30.6]	[5.34]	[1.79]	[0.90]	[0.75]	
Brooding	d_c_	PP					26.3 %
	[82.0]	[18.0]					
Chick-rearing	s(SST)	s(d_c_)	s(depth)	s(PP)	s(d_TZCF_)		39.2 %
	[45.7]	[45.1]	[4.08]	[2.64]	[2.52]		
Black-footed albatross	Model terms [% deviance explained by coefficient]						% deviance explained by model
Incubation	s(SST)	s(d_c_)	SSHa				38.8 %
	[75.9]	[21.9]	[2.18]				
Brooding	s(SST)	d_c_					11.9 %
	[60.5]	[39.5]					
Chick-rearing	s(SST)	s(d_c_)	s(d_TZCF_)	s(EKE)	s(PP)	depth	28.3 %
	[45.5]	[19.8]	[12.2]	[11.2]	[6.44]	[4.84]	

Cubic regression splines retained in the final models are represented by s( ) [84]. SST: sea surface temperature. d_c_: distance to the breeding colony. SSHa: sea surface height anomaly; d_TZCF_: distance to the Transition Zone Chlorophyll Front; EKE: eddy kinetic energy; PP: primary productivity

**Fig. 5:**
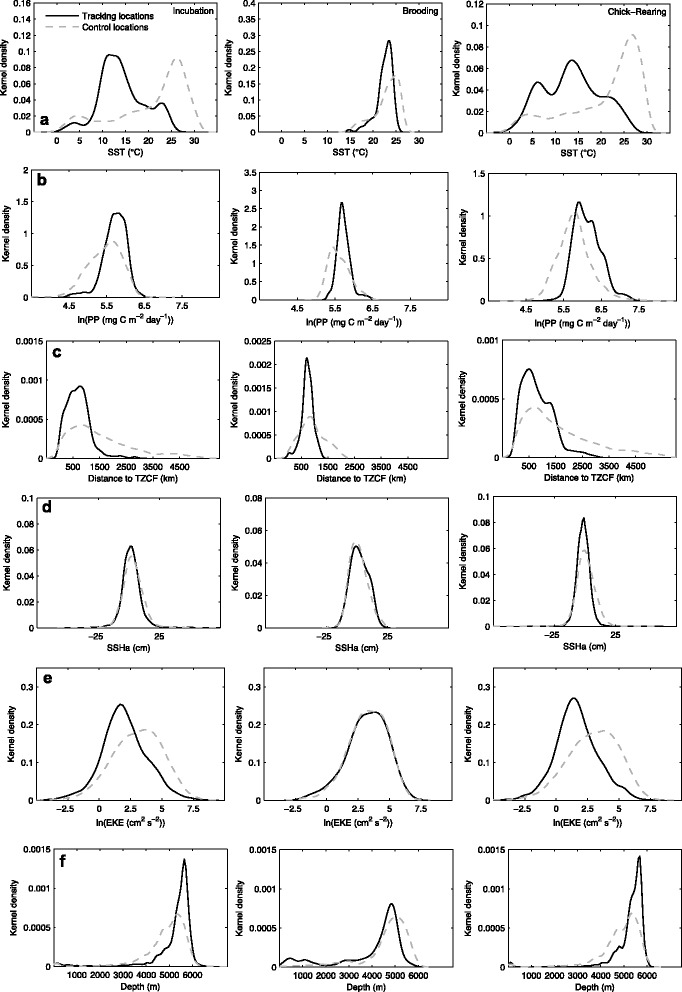
Kernel density of environmental covariates (a-f) at Laysan albatross tracking and control locations. Only environmental covariates retained in final habitat preference models for the species (Table 3) are provided. SST: sea surface temperature; PP: primary productivity; TZCF: Transition Zone Chlorophyll Front; SSHa: sea surface height anomaly; and EKE: eddy kinetic energy

**Fig. 6:**
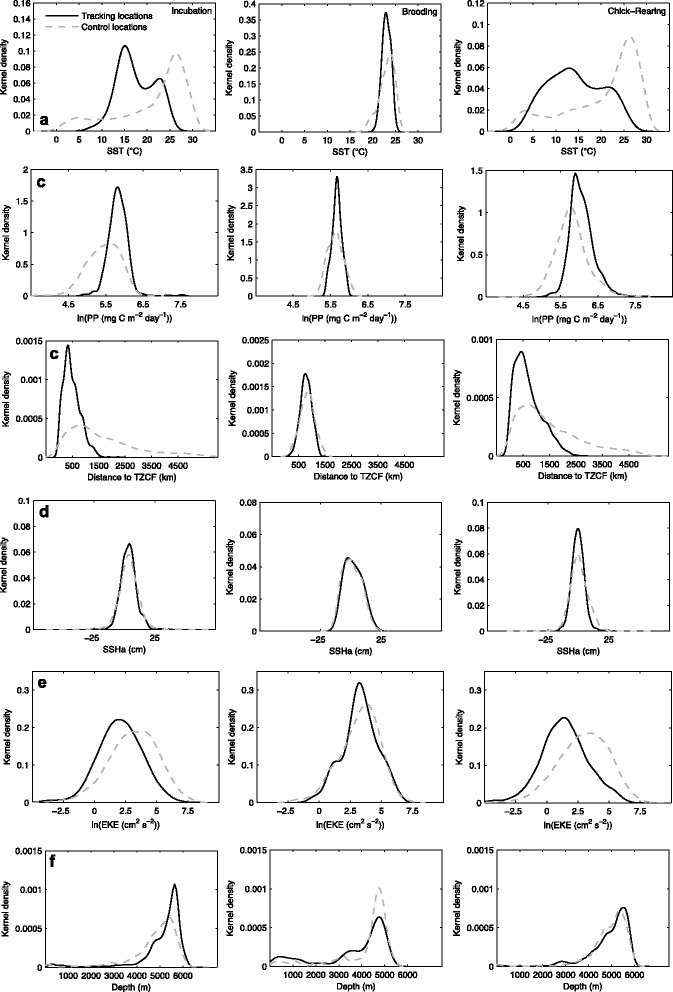
Kernel density of environmental covariates (a-f) at black-footed albatross tracking and control locations. Only environmental covariates retained in final habitat preference models for the species (Table 3) are provided. SST: sea surface temperature; PP: primary productivity; TZCF: Transition Zone Chlorophyll Front; SSHa: sea surface height anomaly; and EKE: eddy kinetic energy

**Fig. 7:**
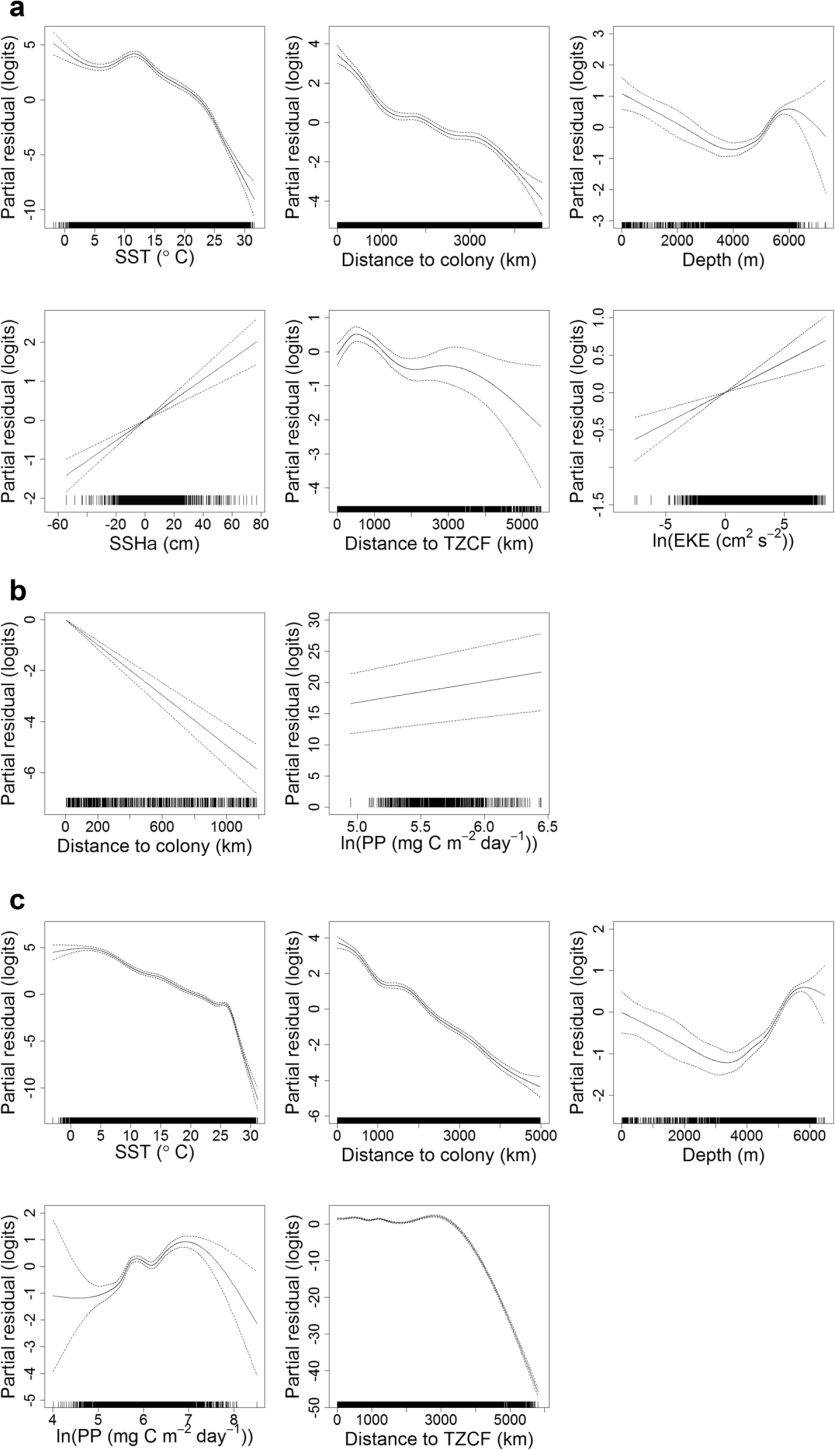
Effects of covariates in final GAMMs for Laysan albatrosses during incubation (a), brooding (b), and chick-rearing (c). The contribution of each retained covariate to the linear predictor is plotted on the scale of the link function (y-axes); the plots can therefore be interpreted as population-level habitat preferences [80]. Dashed lines indicate approximate 95 % confidence intervals. GAMM: generalized additive mixed model; SST: sea surface temperature; SSHa: sea surface height anomaly; TZCF: Transition Zone Chlorophyll Front; EKE: eddy kinetic energy; and PP: primary productivity

**Fig. 8:**
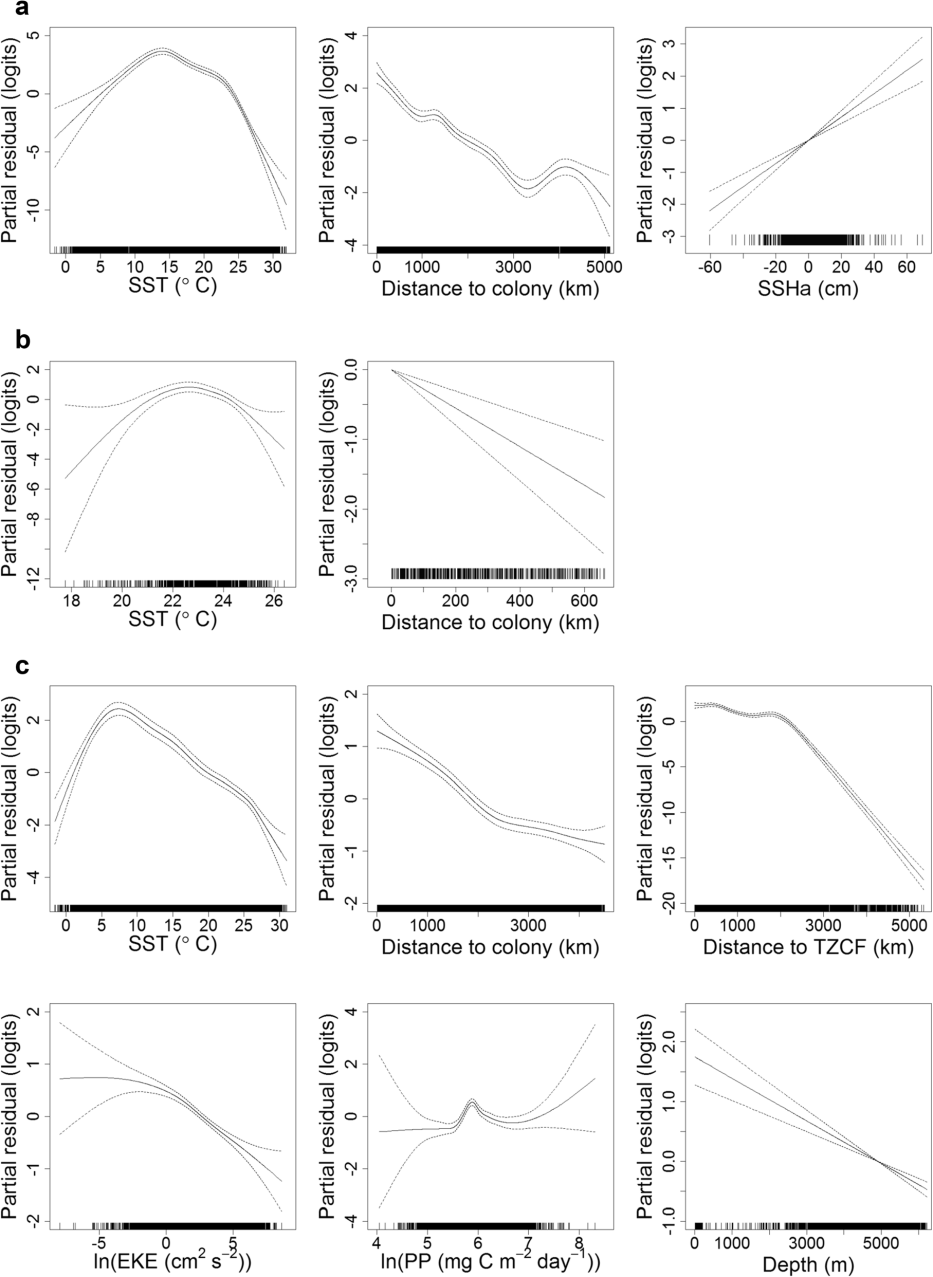
Effects of covariates in final GAMMs for black-footed albatrosses during incubation (**a**) brooding (**b**) and chick-rearing (**c**). The contribution of each retained covariate to the linear predictor is plotted on the scale of the link function (y-axes); the plots can therefore be interpreted as population-level habitat preferences [80]. Dashed lines indicate approximate 95 % confidence intervals. GAMM: generalized additive mixed model; SST: sea surface temperature; SSHa: sea surface height anomaly; TZCF: Transition Zone Chlorophyll Front; EKE: eddy kinetic energy; and PP: primary productivity

The range of accessible thermal habitats was considerably wider during incubation and chick-rearing (~0-31 °C), as compared to brooding for both species (~15-26 °C; Fig. 5a and 6a). Laysan and black-footed albatrosses generally demonstrated a preference for cooler water temperatures compared to availability (Fig. 5a and 6a), especially during incubation and chick-rearing. During these two breeding stages, Laysan and black-footed albatrosses demonstrated differing response curves between SST and habitat preference, after accounting for the effects of other covariates in the final habitat models (Fig. 7a and c; Fig. 8a and c). Laysan albatross preference was highest at a broad range of cool water temperatures (~0-12 °C), decreasing steeply at temperatures greater than ~20 °C (Fig. 7a and c). Black-footed albatross habitat preference was highest at intermediate temperatures, peaking at ~14 °C during incubation and ~7 °C during chick-rearing (Fig. 8a and c).

All final habitat preference models included d_c_, which was retained as a smooth term during incubation and chick-rearing, but as a linear term during brooding for both species (Table 3). In all cases, d_c_ had a negative slope (Fig. 7 and 8), indicating that spatial use of foraging locations decreased at a greater rate than 1/d_c_. Distance to the colony accounted for the greatest proportion of explained deviance in the final habitat model for Laysan albatrosses during brooding, and the second greatest proportion of explained deviance in all other species-stages (20-82 %; Table 3). During incubation and chick-rearing, both Laysan and black-footed albatrosses demonstrated a preference for habitats within ~2000 km of the Tern Island colony (Fig. 7 and 8).

For Laysan albatrosses during brooding, PP was the only environmental covariate retained in the final GAMM; Laysan albatrosses during this stage demonstrated a preference for more productive habitats (Fig. 7b), however, the slope of this relationship was fairly shallow. Generally, a narrower range of primary production was available to foraging albatrosses during brooding (~150-600 mg C m^−2^ day^−1^) as compared to incubation (~30-1000 mg C m^−2^ day^−1^) and chick-rearing; the most productive habitats were available during chick-rearing (~50-2000 mg C m^−2^ day^−1^; Fig. 5b and 6b). During chick-rearing, PP was retained as a smooth term in the final habitat models for both species; preference was generally highest at intermediate levels (~350-1000 mg C m^−2^ day^−1^; Fig. 7c and Fig. 8c), although black-footed albatrosses preference also increased at the highest productivity values (>1350 mg C m^−2^ day^−1^; Fig. 8c).

Laysan albatrosses during incubation and chick-rearing, and black-footed albatrosses during chick-rearing, demonstrated a relationship between d_TZCF_ and habitat preference (Table 3). Preference generally decreased after distances of ~2000-3000 km (Fig. 7a and c; Fig. 8c), indicating that this response may be related to the northerly bias in albatross tracking locations (Fig. 1) rather than a tight association with the TZCF itself. The percent explained deviance for this variable was generally low for Laysan albatrosses (<3 %), but explained 12.2 % of the deviance in the final chick-rearing habitat model for black-footed albatrosses (Table 3).

EKE accounted for the next highest proportion of explained deviance in the black-footed albatross habitat model during chick-rearing (Table 3). Preference was for lower values of EKE (Fig. 8c), contrary to our expectation. EKE was also retained in the final model for Laysan albatrosses during incubation, and demonstrated a positive slope (Fig. 7a), however, this term accounted for a very small proportion of explained deviance (0.75 %; Table 3). SSHa, another indicator of mesoscale activity, was retained in the final incubation models for both species, but accounted for a relatively small proportion of explained deviance (~2 %; Table 3); in both cases preference had a positive relationship with SSHa (Fig. 7a and 8a).

Sea floor depth was retained as a smooth term in the incubation and chick-rearing models for Laysan albatrosses, and as a linear term in the chick-rearing model for black-footed albatrosses (Table 3). Black-footed albatrosses demonstrated a general preference for shallower waters (Fig. 8c), whereas Laysan albatrosses demonstrated a u-shaped response, with a preference for both the shallowest (<2000 m) and deepest habitats (>5000 m; Fig. 7a and c). Sea floor depth accounted for 4-5 % of the explained deviance in final habitat models for these species-stages (Table 3).

## Discussion

### Foraging movements

Temporal changes in foraging behavior have been linked to changing energetic demands of offspring in a variety of central place foragers [23,24,110–112]. Consistent with previous research, Laysan and black-footed albatrosses spent more time at sea and ranged farther from the colony during incubation and chick-rearing than during brooding [26, 28, 29, 32]. Albatrosses have the ability to make long-distance movements during these breeding stages due to the fasting capabilities of adults [incubation spans can last as long as 50 days; [113] and post-guard chicks, which fast between intermittent feeds [40]. During brooding, adults are limited to short trips when young chicks require frequent meals [21].

When conducting long-ranging trips, Laysan albatrosses generally foraged at more northerly latitudes compared to black-footed albatrosses. Ommastrephid squid, which comprise the largest proportion of the Laysan albatross diet, also have a more northerly distribution in the NPTZ compared to flying fishes [90], the eggs of which comprise the largest proportion of the black-footed albatross diet [86]. During chick-rearing, black-footed albatrosses traveled farther north and east than during incubation, reaching greater distances than Laysan albatrosses. The northward shift may be a response to seasonal changes in the distribution of prey associated with the NPTZ [50]; the eastward shift is likely a response to prey aggregations associated with productive coastal upwelling along the western coast of North America during this time of year [114, 115]. Although flying fish eggs dominate the diet of black-footed albatrosses during chick-rearing [86], we expect that this reflects foraging events in subtropical waters, and that squid and other prey types would be consumed by black-footed albatrosses foraging in the California Current.

### Interspecific habitat segregation

We found that Laysan and black-footed albatrosses demonstrated spatial segregation of core and focal foraging areas during the incubation and chick-rearing periods, which agrees with previous studies conducted during both the breeding [28, 29, 32] and non-breeding periods [116]; Shaffer et al., unpublished data]. However, habitat segregation between sympatric species can vary depending on reproductive stage, and the strength of these differences may be affected by prey availability, energetic constraints, or changes in interspecific encounters [117–120]. During brooding, when reproductive constraints restrict movements, overlap of core and focal foraging areas was greatest [see also [29]. Although our results showed that overall foraging ranges (95 % UD) were spatially segregated during brooding, this was likely due to a few longer-distance trips made by Laysan albatrosses, reflected in the high variability of maximum distances reached during brooding (Table 1).

Greater dietary partitioning during brooding could provide a means of reducing competitive interactions due to shared feeding areas. There is limited information, however, on changes in diet throughout the reproductive period in these species; in other albatross species, diet can change considerably over the course of the breeding season [121]. The most comprehensive dietary dataset for Hawaiian albatrosses are mainly representative of the chick-rearing period and are not separated for each stage [86]. Other diet studies have focused on by-caught birds in fisheries [87] or analysis of regurgitated pellets from chicks during the rearing period only [122]. Comparison of a limited number of chick diets from the brooding period suggests some dietary segregation during this stage; squid and fish were found more frequently in the diet of Laysan albatross chicks, and flying fish eggs were found more frequently in the diet of black-footed albatross chicks (M.A. Kappes, unpublished data).

### Activity patterns at sea

Contrary to our expectation, neither species changed the frequency of landings on the sea surface during the brooding period to meet higher energy requirements during this stage. Although black-footed albatrosses landed more frequently on average during brooding, differences were not statistically significant due to high variability in landing rate during this stage (Table 2). Laysan albatrosses did, however, spend a greater proportion of time in flight during brooding as compared to the incubation and chick-rearing periods. Weimerskirch et al. [123] suggests that periods of sustained movement in wandering albatrosses is an adaptation to locating dispersed and unpredictable prey, a strategy which may be relevant to Hawaiian albatrosses foraging in a warm, oligotrophic environment during brooding.

The brooding period is also when core foraging areas of Laysan and black-footed albatrosses overlap the most, so species differences in timing of foraging activities could minimize competition for prey resources during this stage. It has been proposed that higher levels of rhodopsin in the eyes of Laysan albatrosses make them better adapted to nocturnal feeding than black-footed albatrosses [86]. Consistent with this idea, we found that Laysan albatrosses landed more frequently at night than black-footed albatrosses during all breeding stages, but both species actively foraged at night. Overall, there was a bias towards foraging during daylight hours for both species, which is supported by other studies [124, 125]. During brooding, time in flight and daytime landing rates were similar between species, and although landing rates differed at night, this was the case during all breeding stages. Therefore, we did not find evidence that Laysan and black-footed albatrosses changed their behavior during brooding as a means of reducing competitive interactions when spatial overlap was greatest [126].

Hawaiian albatrosses generally spend more time in flight [74-91 %; current study, [124], particularly during the brooding period, than other albatross species [44-69 %; [127, 128]. This may be adaptive for foraging in an oligotrophic environment, where prey resources are likely unpredictable [123]. Tropical seabirds that feed in lower productivity environments generally have a greater flight proficiency to allow travel between dispersed prey patches [38]. Available research on energetics of Laysan albatrosses does not suggest lower energy expenditure rates compared to other albatross species during the incubation period [129], however, a comparison to energy expenditure rates during brooding could provide insight as to whether increased time in flight reduces energetic costs when foraging in a low-productivity environment.

### Habitat preference

We combined satellite tracking data with geolocation data when modeling habitat preference, in order to obtain coverage of the entire breeding period. However, this could lead to potential biases given the different accuracies of each tag type (satellite tags are accurate to <10 km [53]; geolocation tags are accurate to ~200 km [54]). To address this potential bias, we investigated the effects of tag type on habitat models by comparing intercept-only GAMMs for each species and reproductive stage to models including tag type as a candidate variable. Because AIC values were not improved by the addition of this term, differences in tag accuracy should not have affected results of habitat preference modeling. We also did not find an effect of gender on habitat preference, which agrees with previous studies of these species [28, 29, 130].

SST accounted for much of the variability in final habitat models during incubation and chick-rearing, when both species preferentially selected cooler waters compared to availability. Similarly, when using a different technique to model habitat use of Hawaiian albatrosses during incubation, Kappes et al. [32] found that SST was the most important environmental variable related to first passage time [131], a measure of area-restricted search [132]. Overall, black-footed albatrosses preferred slightly warmer marine habitats than Laysan albatrosses; this likely relates to differences in diet and spatial separation of prey resources along the latitudinal SST gradient in the NPTZ [86, 90].

We hypothesized that each species would use the same environmental covariates to select habitats throughout the breeding season, but that reproductive constraints and seasonal cycles in marine habitats would influence the composition of utilized habitats. SST accounted for the greatest proportion of variability in black-footed albatross habitat models during all reproductive stages and for Laysan albatross habitat models during incubation and chick-rearing; however, SST was not retained in the final habitat model for Laysan albatrosses during brooding. PP was the only environmental covariate retained in the brooding habitat model for Laysan albatrosses, but the majority of variability in habitat preference was explained by d_c_. Both species demonstrated less distinct habitat preferences during brooding; preference increased only slightly with PP for Laysan albatrosses, and black-footed albatrosses had a smaller peak in preference in relation to SST compared to incubation and chick-rearing. This reflects the narrower range of environmental conditions available for selection during brooding, and helps explain why model goodness of fit was lower during brooding than during other breeding stages (Table 3).

Given the constraint of central place foraging during all stages of breeding, we included d_c_ as a candidate covariate when modeling habitat preference. All final habitat models included d_c_, suggesting that accessibility decreased at a rate greater than 1/d_c_ [80–82], and that the null usage model could be further refined. The inclusion of d_c_ in final habitat models could also indicate a preference for resources closer to the colony that are not linked to the covariates examined. Albatrosses use gliding flight on sea surface winds for locomotion [98], so an important consideration may be the effect of wind on habitat accessibility [133–136]. Because Hawaiian albatrosses tend to avoid facing headwinds during long-distance movements [137], accounting for differences in wind direction might improve the null usage model. Overall accessibility of habitats may be affected by prevailing winds, but the fact that wind variables were not retained in final habitat models suggests that Laysan and black-footed albatrosses do not select foraging locations based on wind conditions. Alternatively, wind variables examined may not sufficiently represent the complex manner in which albatrosses use surface winds.

Together, SST and d_c_ accounted for most of the explained deviance in final habitat models for both species. For black-footed albatrosses during chick-rearing, d_TZCF_ and EKE also accounted for considerable (>10 %) proportions of explained deviance. Habitat preference declined after distances of ~2000 km from the TZCF, indicating that this response may be related to the northerly bias in tracking locations rather than a tight association with the TZCF itself. Although we expected that a higher degree of mesoscale activity would enhance foraging opportunities, black-footed albatrosses preferred habitats with lower EKE during chick-rearing. Their use of the pelagic northeast Pacific, a region of relatively low EKE [138], suggests that black-footed albatrosses do not rely on prey associated with high eddy activity during the chick-rearing period; leatherback turtles (*Dermochelys coriacea*) in the North Pacific also demonstrate a negative relationship between habitat use and EKE [139]. However, low accuracy of geolocation data may have limited our ability to detect responses to mesoscale activity.

The environmental covariate with the greatest influence on habitat preference of Laysan and black-footed albatrosses was SST. Sea floor depth, SSHa, PP, d_TZCF_, and EKE were also retained as covariates in final habitat models, however, these terms represented a smaller proportion of explained deviance. This suggests that after accounting for SST preference, the location of large-scale fronts (d_TZCF_), mesoscale activity (EKE and SSHa), and bathymetric domain do not have a strong influence on habitat selection of Hawaiian albatrosses. Other environmental covariates describing frontal characteristics (dySST) and wind-driven oceanic upwelling or downwelling (wind stress curl) were not retained in final models, despite characterizing physical features or processes that could drive prey availability for Hawaiian albatrosses [88–91,93]. Studies of other marine top predators have also found the greatest behavioral responses to SST compared to other marine habitat variables (Simmons et al., 2007; Teo et al., 2007). Environmental covariates examined might also be improved by incorporating time lags, as the response of prey resources may trail behind increases in productivity.

### Breeding in an oligotrophic marine environment

**Fig. 9:**
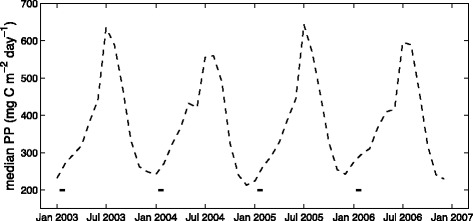
Primary productivity (PP) near the Tern Island colony. Median monthly PP was calculated within the mean foraging range of Laysan and black-footed albatrosses during brooding (370 km); brooding periods are indicated by solid lines at the bottom of the figure

Laysan and black-footed albatrosses differ from other albatross species in that they breed in an oligotrophic marine environment [140, 141]. They leave this environment during incubation and chick-rearing to forage in cooler, more northern waters, but are restricted to a low-productivity environment during brooding when energy requirements are greatest [21]. For wandering albatrosses, the brooding period coincides with seasonal increases in prey abundance in the Southern Ocean [15]. Little is known about temporal variability of prey consumed by Laysan and black-footed albatrosses, but it has been suggested that these species breed during winter to match peaks in prey abundance [142], or alternatively to avoid the period of maximum summer heat [113]. PP near the Tern Island colony is lower during brooding compared to the rest of the year (Fig. 9), so assuming PP is a good indicator of prey abundance, local prey resources may in fact be limited during the brooding period. Timing of breeding may instead be related to the relative proximity of preferred thermal habitats; the latitudinal position of the TZCF, and the cooler waters associated with it, are closest to the Tern Island colony during the brooding and early chick-rearing periods [50].

Body mass of adult Laysan albatrosses declines throughout the breeding season, from the start of the incubation period until the second half of the chick-rearing period [143]. This suggests a high cost of central place foraging from breeding sites in the Hawaiian Islands; other albatross species do not demonstrate equivalent declines in adult mass during breeding [40, 144, 145]. The change to positive mass gain in mid-May may be related to seasonal increases in PP close to the colony (Fig. 9) and the closer proximity of preferred habitats of the NPTZ at this time [50]; the shorter commuting distance to preferred habitats could help adults restore body condition.

During chick-rearing, many Procellariform species alternate between short foraging trips that maximize energy delivery to young, with long trips that restore adult condition [146, 147]; this is reflected in higher food delivery rates after short trips compared to long trips [reviewed by [148]. Previous research has demonstrated that Hawaiian albatrosses mix short and long trips during chick-rearing [28, 29], though we did not observe strong bimodality in foraging trip duration during this stage (Fig. 3b). Contrary to expectation, Laysan albatrosses fledge chicks in better condition when they perform fewer short trips for every long trip during chick-rearing [149]. This suggests that shorter trips are less profitable, and that longer trips to preferred habitats of the NPTZ are required for successful chick-rearing. It is unclear, however, to what extent low food delivery rates affect chick survival. Along with food shortages, storm events and nest desertion are common causes of chick mortality [150, 151].

In addition to seasonal changes, the latitudinal position of the TZCF varies interannually, particularly in response to El Niño-Southern Oscillation events [50]. When the wintertime position of the TZCF is significantly north of its seasonal norm, Laysan and black-footed albatrosses experience dramatic breeding failures [152, 153]. Laysan albatrosses also demonstrate lower reproductive success and mass gains in years when they range farther and spend longer durations at sea [32]. This suggests that changes in the proximity of preferred habitats during breeding can lead to demographic effects in these species. Changes in SST in the North Pacific due to global climate change [154, 155] will likely have considerable impacts on Hawaiian albatrosses, given the importance of SST in foraging habitat selection. In addition, poleward shifts in westerly winds associated with climate change [156–158] may lead to changes in the positioning of the North Pacific Current [52] and access to preferred habitats. In the Southern Ocean, the poleward shift of the westerlies has positively affected breeding performance of wandering albatrosses, but a continuing shift may make the location of breeding colonies less optimal in the future [159]. A northward shift in the positioning of the TZCF due to climate change could have negative effects on Laysan and black-footed albatross populations, if preferred habitats become more distant from the colony during critical portions of the breeding season. Comparisons among breeding locations within the Hawaiian islands would provide insight into these interactions [160], particularly at Midway Atoll and Laysan Island, where the greatest numbers of breeding pairs occur for both species [151].

## Conclusions

For central place foragers, the location of suitable breeding sites can have large effects on behavior and habitat use. Because Hawaiian albatrosses breed in an oligotrophic marine environment, they forage in lower-productivity waters than other albatross species during the energetically costly brooding period. They also spend a greater proportion of time in flight, a strategy that may be adaptive for locating unpredictable food resources. Additional research on the foraging energetics of these species could help resolve how Hawaiian albatrosses meet energetic demands during brooding, when spatial overlap between species is greatest and primary productivity near the colony is at an annual low. During incubation and chick-rearing, Laysan and black-footed albatrosses make long distance movements north of the colony, selecting cooler water temperatures and making use of the regionally productive NPTZ. A poleward shift in the TZCF due to climate change could negatively affect Laysan and black-footed albatross populations, given breeding failures in years when the TZCF is shifted significantly farther north. A comprehensive analysis of Hawaiian albatross foraging behavior and reproductive success in relation to the position of the TZCF will be necessary to predict population-level effects of climate change.

### Availability of supporting data

The tracking datasets supporting the results of this article are available by request from the BirdLife Tracking Ocean Wanderers database, [http://seabirdtracking.org].

## Additional file

Additional file 1:
**Appendix A Laysan and** **black-footed** **albatross habitat preference modeling using an alternative null usage model.** Habitat preference was modeled using a more restrictive null model of usage to reflect the northern bias of tracking locations; for each species-stage, minimum convex polygons of tracking locations were used to limit the southern extent of control locations, and maximum observed ranges were used to limit the northern extent of control locations. (DOCX 25 kb)

## Abbreviations

d_c_: Distance to the colony
d_TZCF_: Distance to the Transition Zone Chlorophyll Front
dySST: Latitudinal gradient in sea surface temperature
EKE: Eddy kinetic energy
GAMM: Generalized additive mixed model
NPTZ: North Pacific Transition Zone
PP: Primary productivity
SSHa: Sea surface height anomaly
SST: Sea surface temperature
TZCF: Transition Zone Chlorophyll Front
UD: Utilization distribution
