# Supplementary material for: Reproductive constraints influence habitat accessibility, segregation, and preference of sympatric albatross species
Source: Mov Ecol. 2015 Sep 29;3:34. doi: 10.1186/s40462-015-0063-4 (PMC4587674; doi:10.1186/s40462-015-0063-4)
Supplement: Additional file 1: — Appendix A Laysan and black-footed albatross habitat preference modeling using an alternative null usage model. Habitat preference was modeled using a more restrictive null model of usage to reflect the northern bias of tracking locations; for each species-stage, minimum convex polygons of tracking locations were used to limit the southern extent of control locations, and maximum observed ranges were used to limit the northern extent of control locations. (DOCX 25 kb) [file 40462_2015_63_MOESM1_ESM.docx]

**Appendix A Laysan and black-footed albatross habitat preference modeling using an alternative null usage model.**  Habitat preference was modeled using a more restrictive null model of usage to reflect the northern bias of tracking locations; for each species-stage, minimum convex polygons of tracking locations were used to limit the southern extent of control locations, and maximum observed ranges were used to limit the northern extent of control locations.**Table A1 Results of GAMM model selection using an alternative null usage model.** Final Laysan and black-footed habitat preference models were arrived at by forward selection based on AIC using a subset of individuals, followed by cross-validation using the remaining individuals. Cubic/cyclic regression splines retained in the final models are represented by s( ) [1]. GAMM: generalized additive mixed model; SST: sea surface temperature; d_c_: distance to the breeding colony; SSHa: sea surface height anomaly; dySST: latitudinal sea surface temperature gradient; curl: wind stress curl; wind: wind direction; EKE: eddy kinetic energy; d_TZCF_: distance to the Transition Zone Chlorophyll Front; PP: primary productivity.

**Table A1**

| **Laysan albatross** | **Model terms [% deviance explained by coefficient]** | | | | | | | | | | **% deviance**  **explained by model** |
| --- | --- | --- | --- | --- | --- | --- | --- | --- | --- | --- | --- |
| Incubation | s(SST) | | s(d_c_) | s(depth) | s(SSHa) | dySST | s(curl) | s(wind) | EKE | s(d_TZCF_) | 23.9% |
|  | [47.6] | | [37.0] | [7.02] | [4.98] | [1.36] | [0.95] | [0.44] | [0.39] | [0.32] |  |
| Brooding | d_c_ | |  |  |  |  |  |  |  |  | 10.4% |
|  | [100] | |  |  |  |  |  |  |  |  |  |
| Chick-rearing | s(d_c_) | | s(SST) | s(depth) | s(PP) | s(SSHa) | s(d_TZCF_) |  |  |  | 24.2% |
|  | [53.0] | | [35.7] | [4.54] | [3.42] | [1.71] | [1.57] |  |  |  |  |
| **Black-footed albatross** | | **Model terms [% deviance explained by coefficient]** | | | | | | | | | **% deviance**  **explained by model** |
| Incubation | s(SST) | | d_c_ | s(depth) | SSHa |  |  |  |  |  | 23.4% |
|  | [71.2] | | [19.7] | [6.35] | [2.75] |  |  |  |  |  |  |
| Brooding | d_c_ | | s(SST) |  |  |  |  |  |  |  | 7.09% |
|  | [51.2] | | [48.8] |  |  |  |  |  |  |  |  |
| Chick-rearing | s(SST) | | s(d_TZCF_) | EKE | PP | SSHa | s(curl) | s(wind) | s(d_c_) | s(depth) | 12.5% |
|  | [38.9] | | [23.3] | [16.3] | [4.37] | [4.13] | [3.99] | [3.12] | [3.06] | [2.77] |  |

**Figure A1 Randomly generated control locations using an alternative null model of usage.** During the incubation **(a)**, brooding **(b)**, and chick-rearing **(c)** periods, minimum convex polygons of Laysan and black-footed albatross tracking locations were used to limit the southern extent of control locations, and maximum observed ranges were used to limit the northern spatial extent of control locations. Control locations were selected at a rate proportional to accessibility (distance to the Tern Island colony (d_c_)^-1^).

**Figure A2 Effects of covariates in final GAMMs for Laysan albatrosses using an alternative null usage model**. For the incubation **(a)**, brooding **(b)**, and chick-rearing **(c)** periods, the contribution of each retained covariate to the linear predictor is plotted on the scale of the link function (y-axes); the plots can therefore be interpreted as population-level habitat preferences [2]. Dashed lines indicate approximate 95% confidence intervals. GAMM: generalized additive mixed model; SST: sea surface temperature; SSHa: sea surface height anomaly; dySST: latitudinal sea surface temperature gradient; EKE: eddy kinetic energy; TZCF: Transition Zone Chlorophyll Front; PP: primary productivity.

**Figure A2**


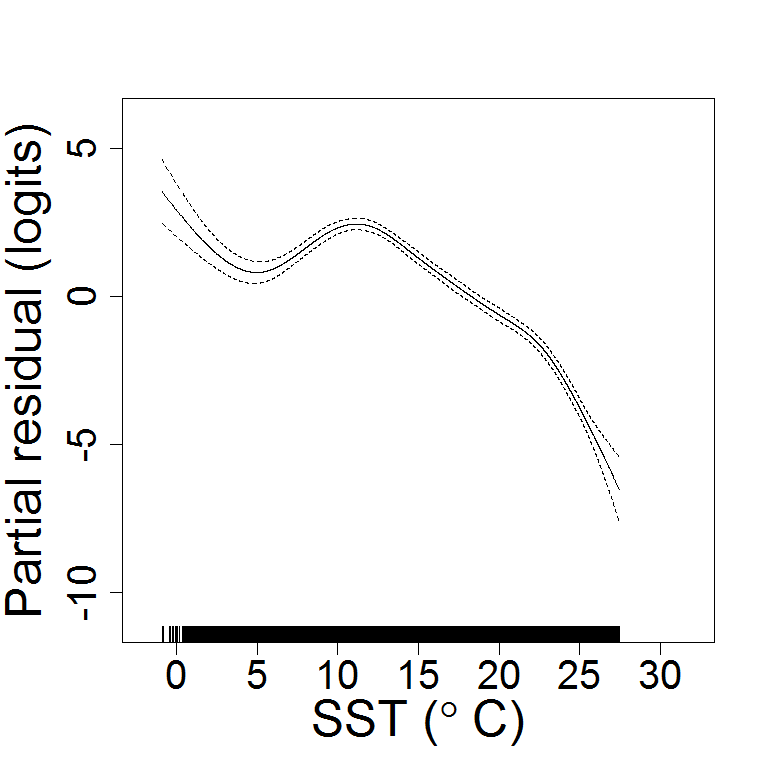

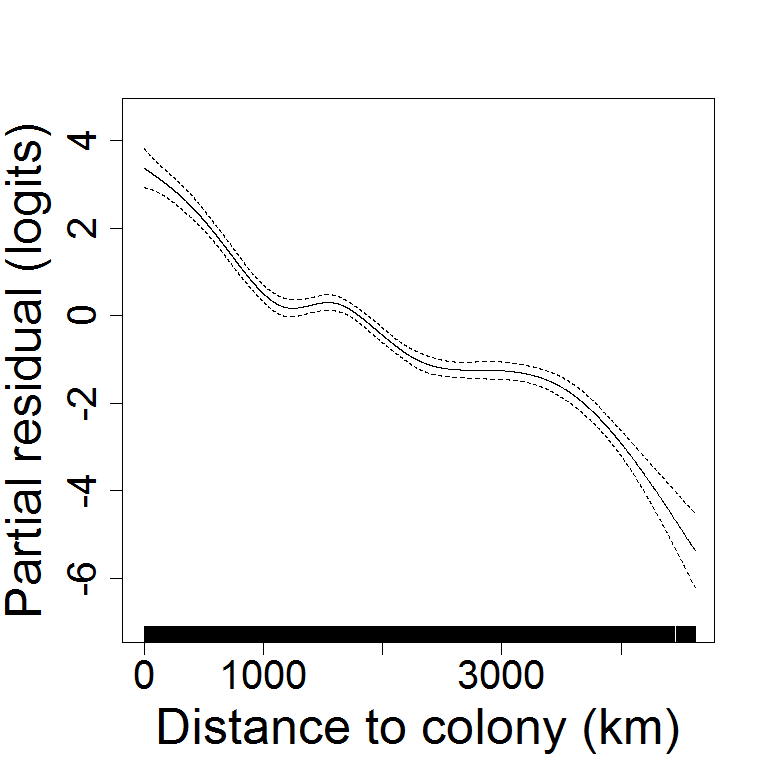

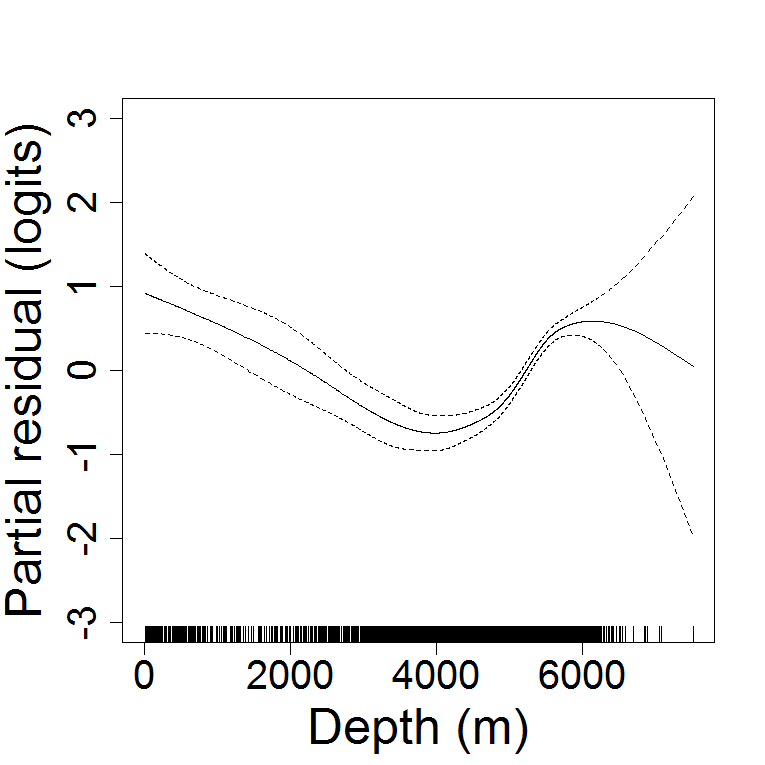


a


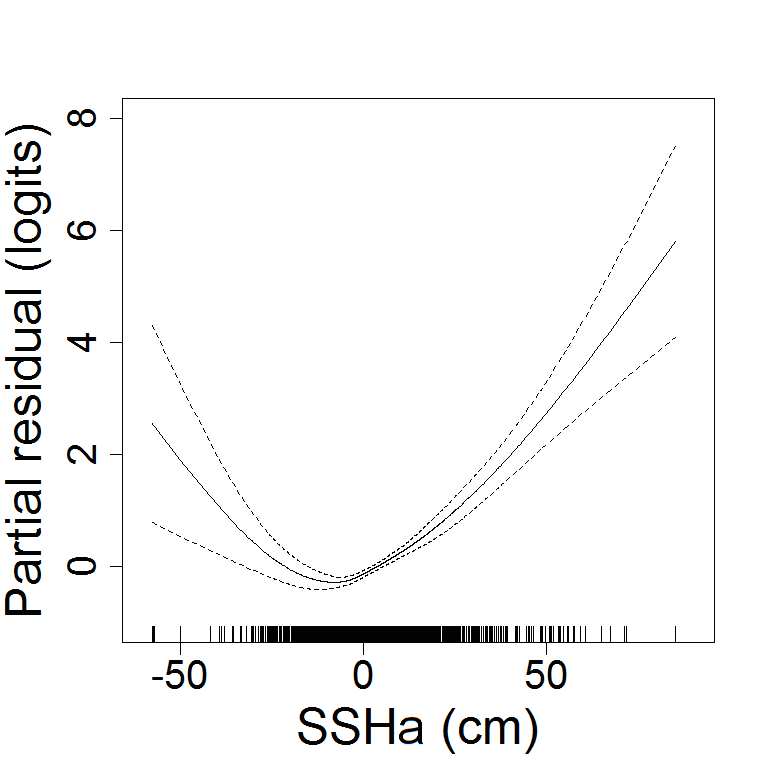

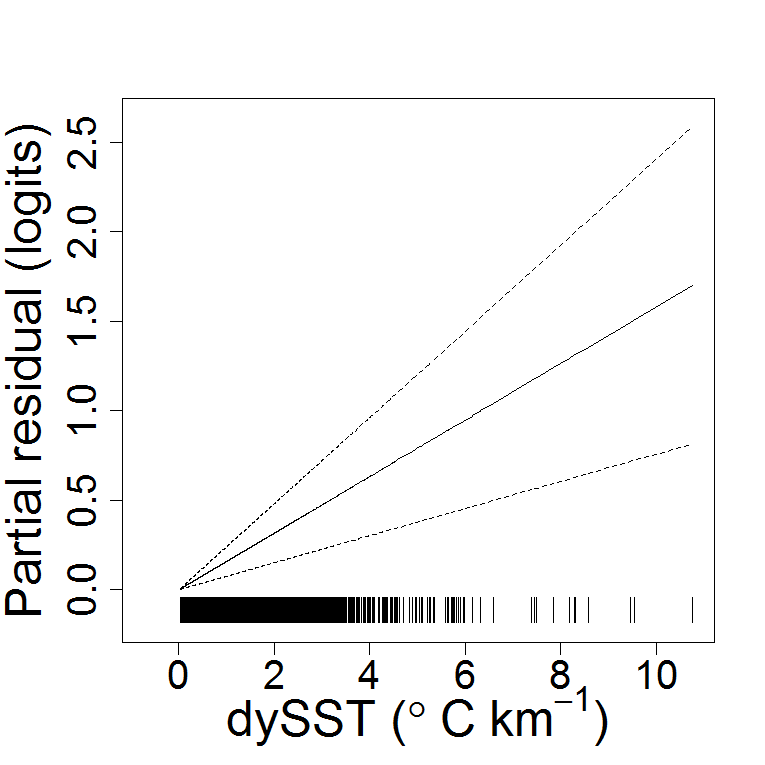

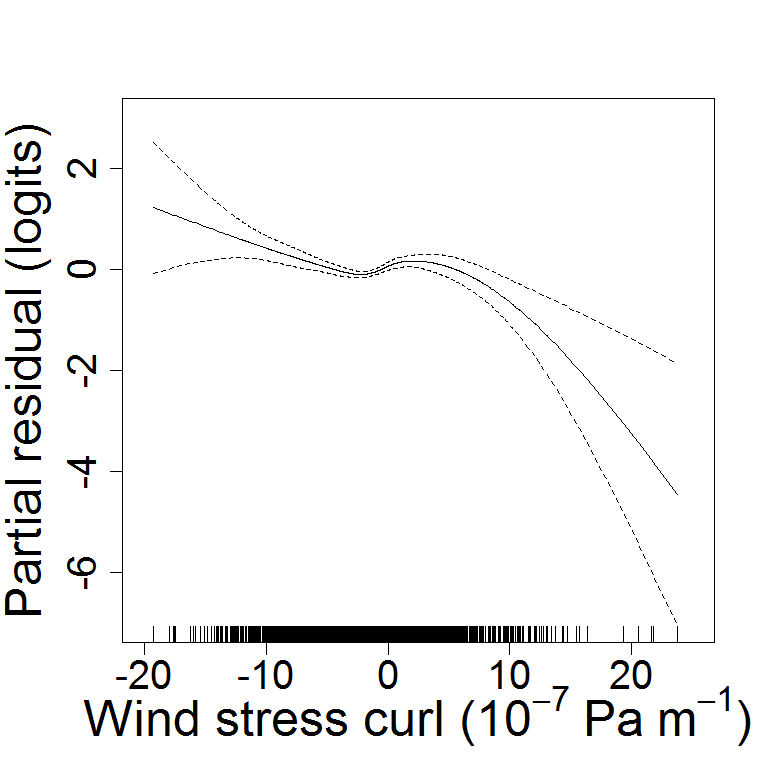


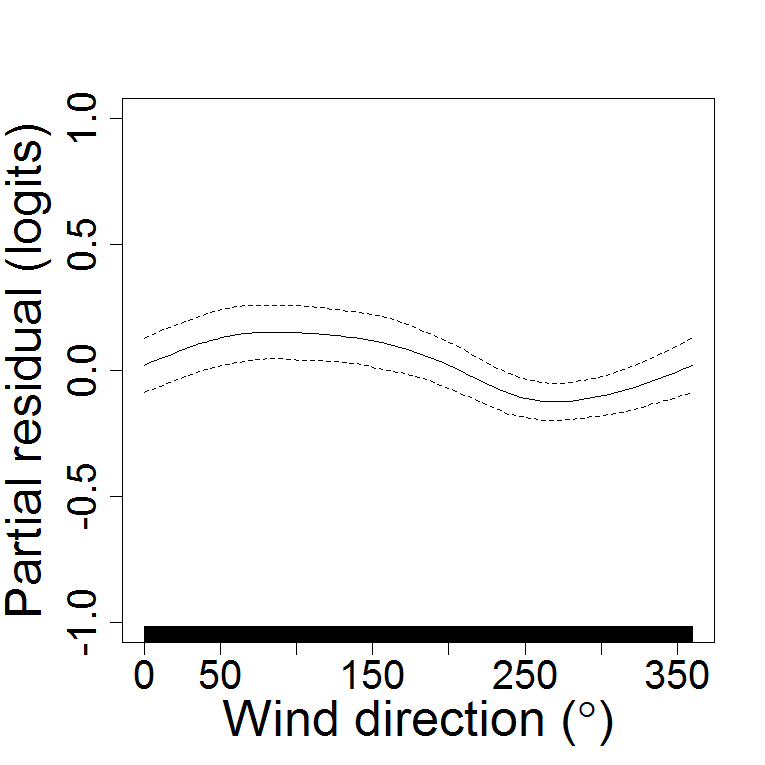

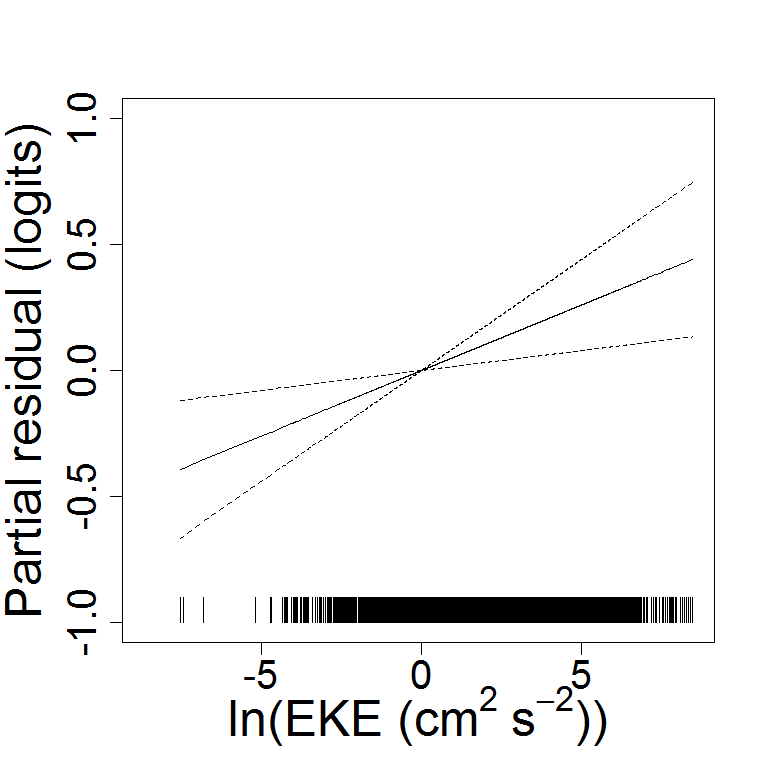

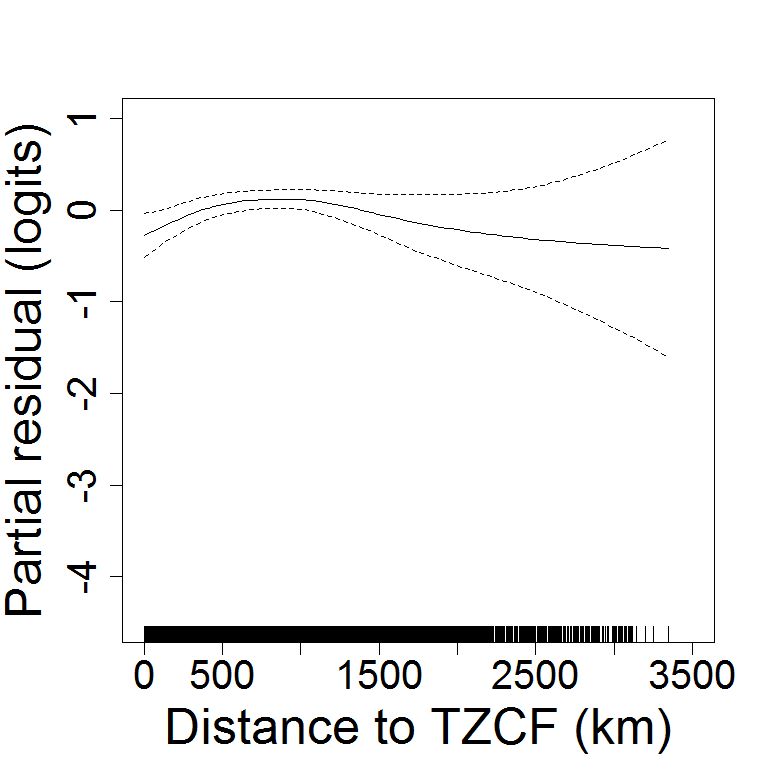


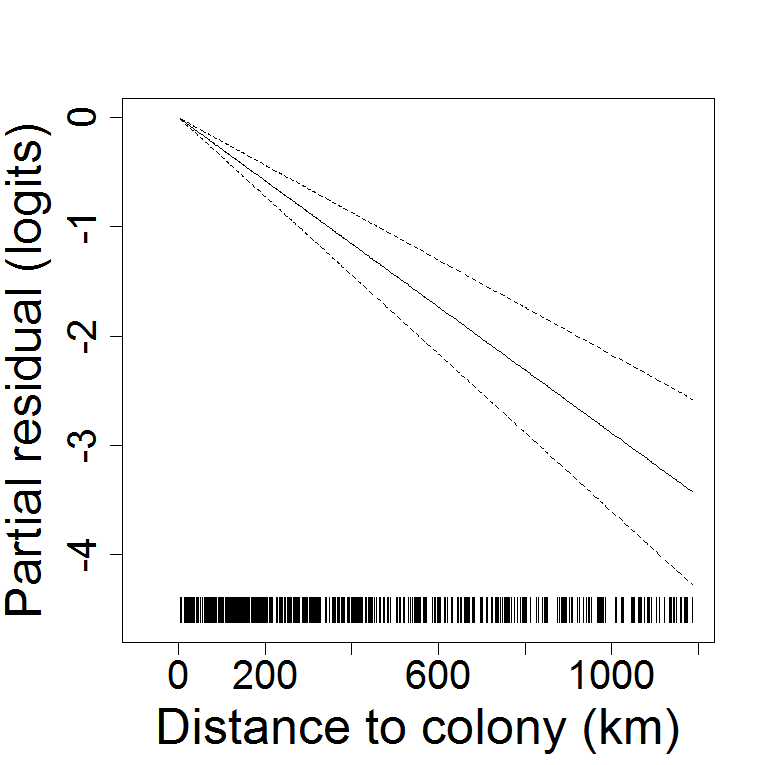


b

**Figure A2** (cont’d)


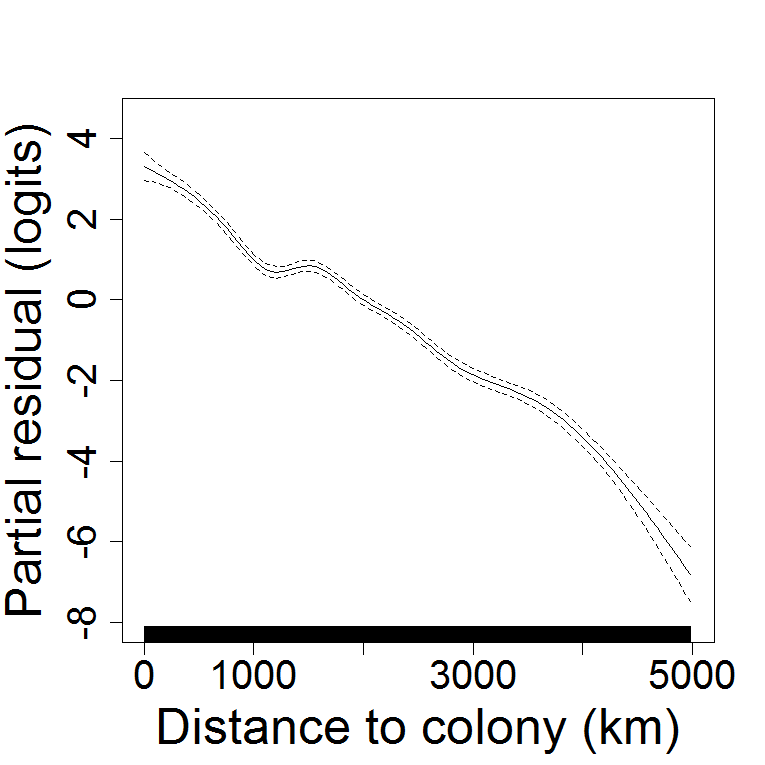

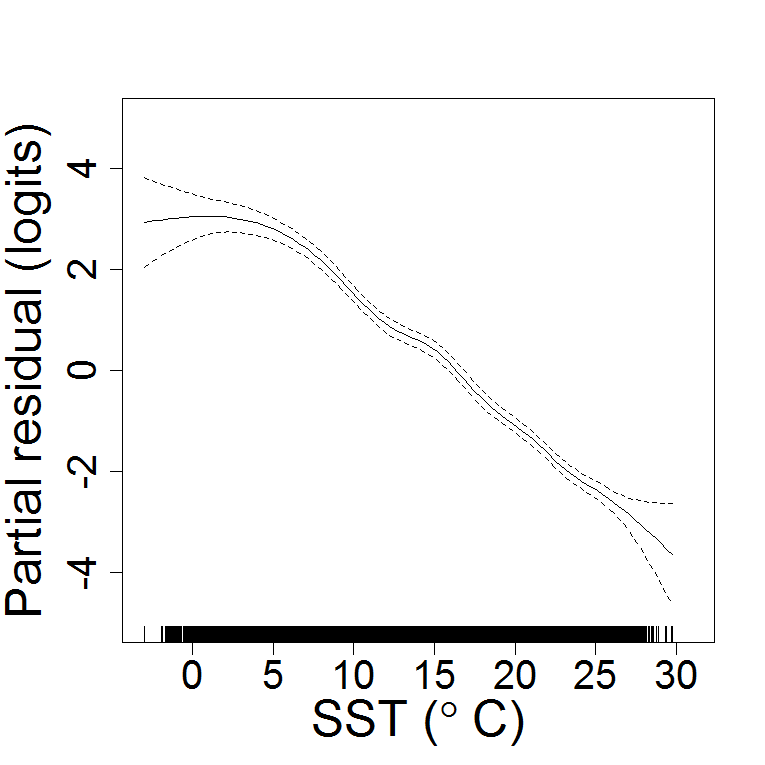

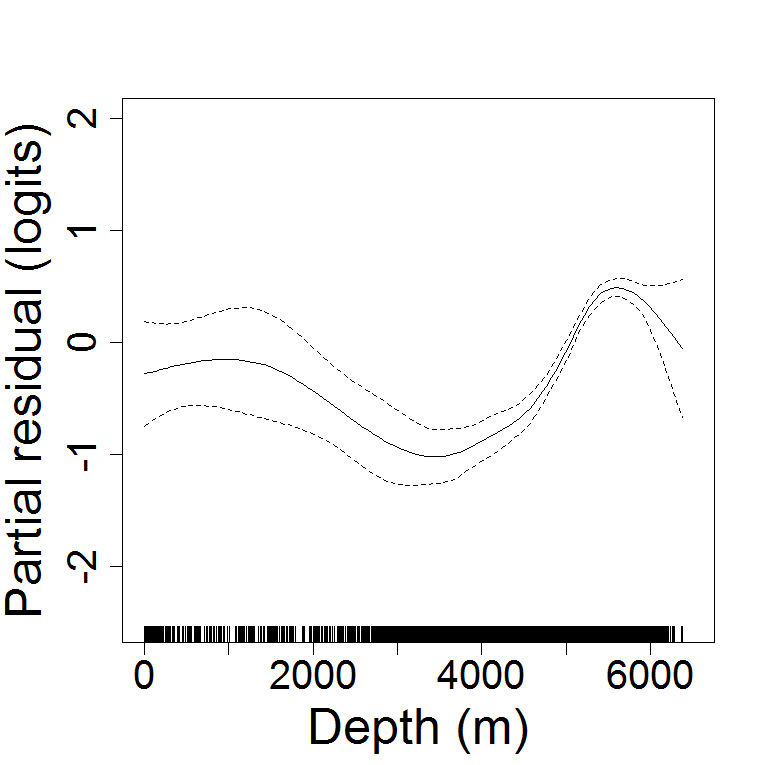


c


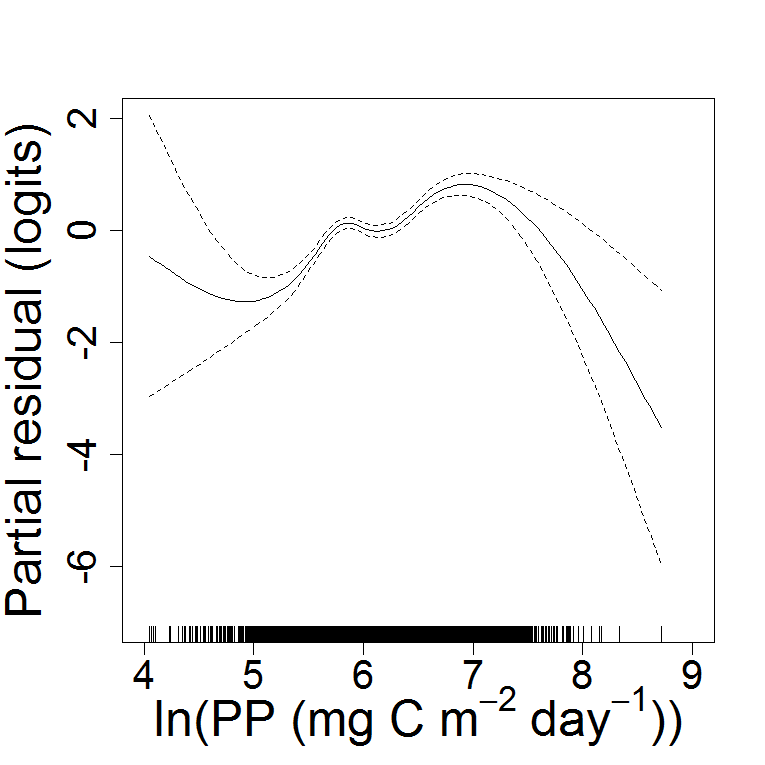

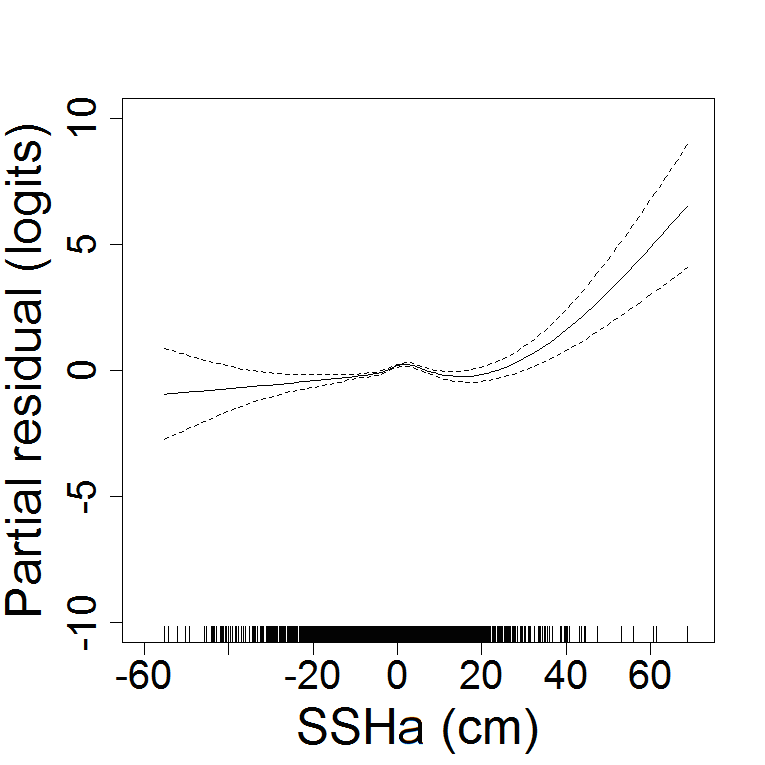

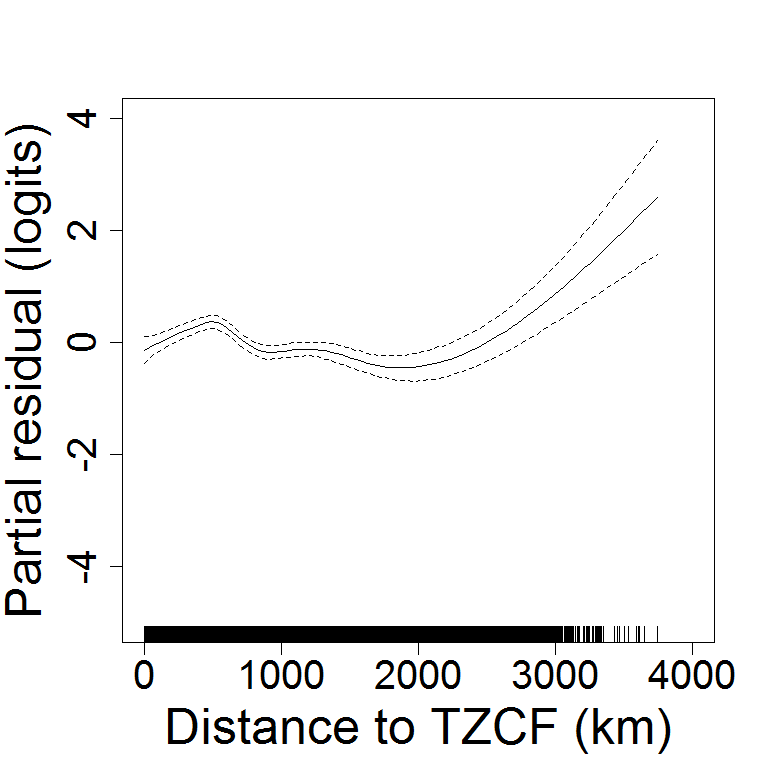
**Figure A3 Effects of covariates in final GAMMs for black-footed albatrosses using an alternative null usage model**. For the incubation **(a)**, brooding **(b)**, and chick-rearing **(c)** periods, the contribution of each retained covariate to the linear predictor is plotted on the scale of the link function (y-axes); the plots can therefore be interpreted as population-level habitat preferences [2]. Dashed lines indicate approximate 95% confidence intervals. GAMM: generalized additive mixed model; SST: sea surface temperature; SSHa: sea surface height anomaly; TZCF: Transition Zone Chlorophyll Front; EKE: eddy kinetic energy; PP: primary productivity.


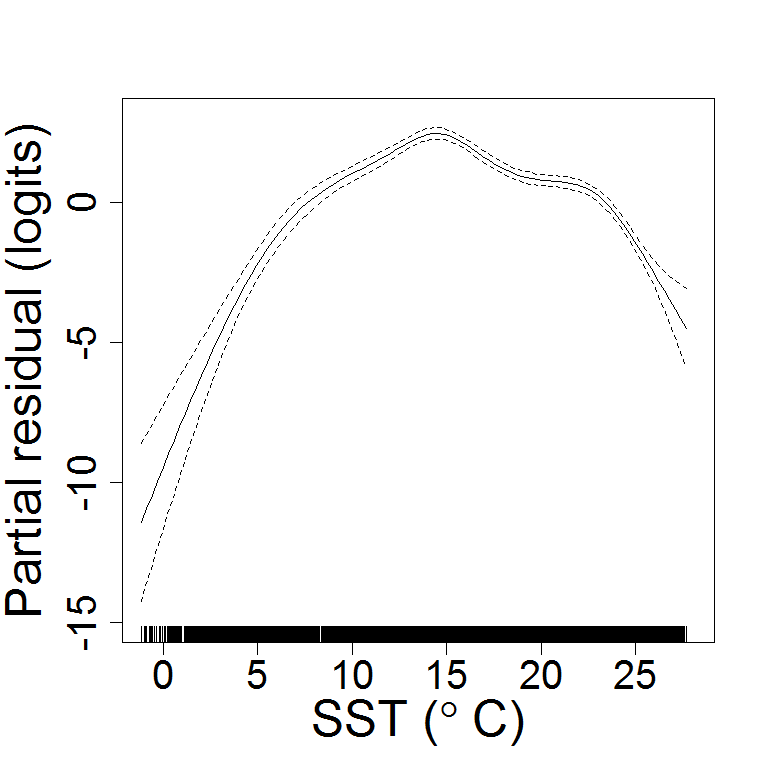

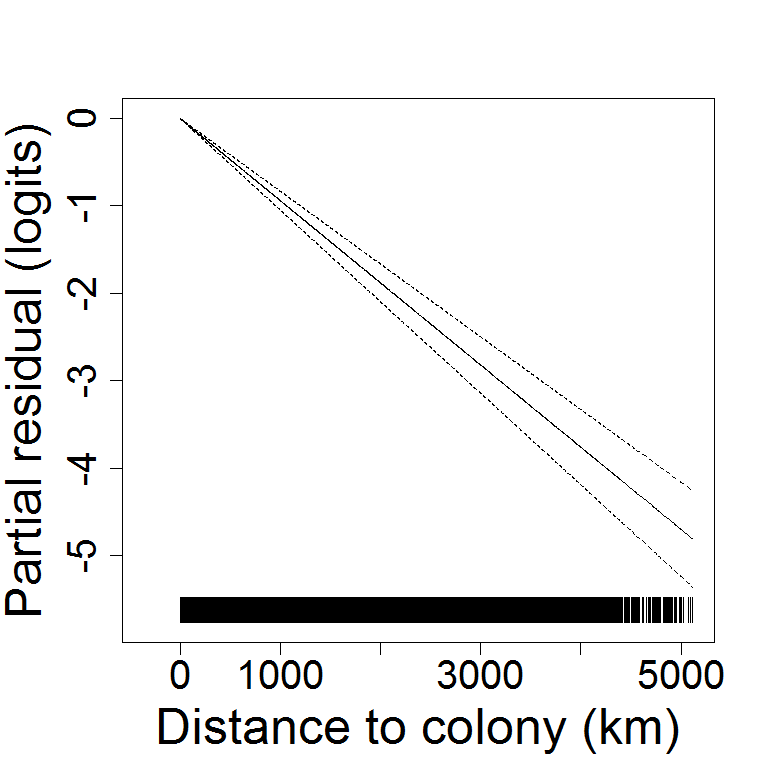

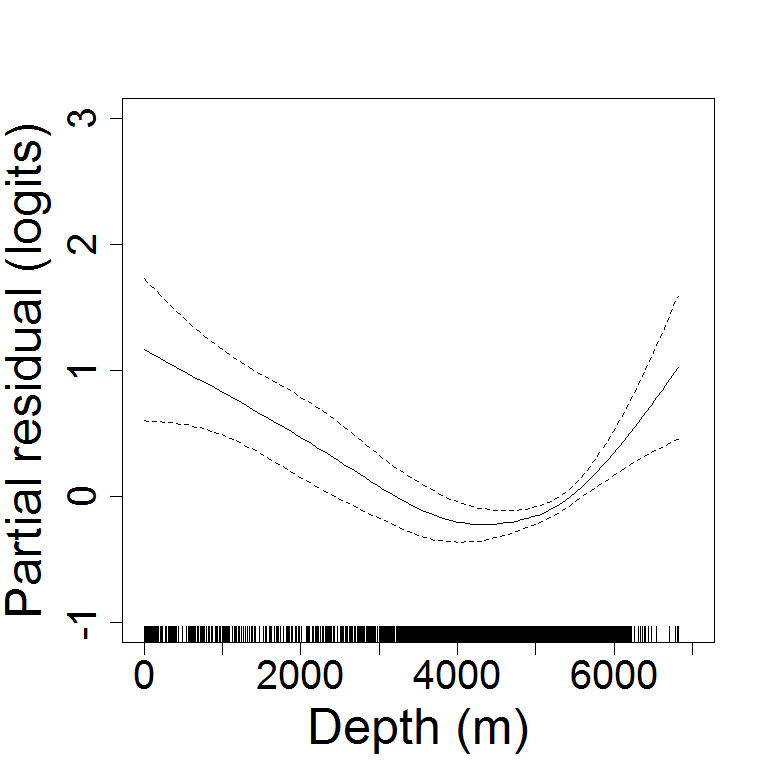


a


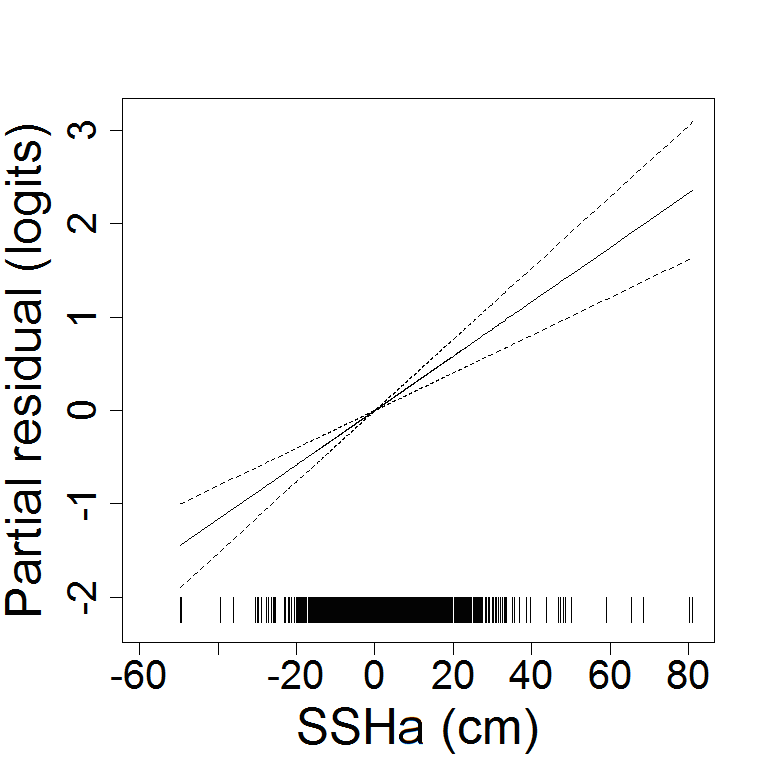


**Figure A3** (cont’d)


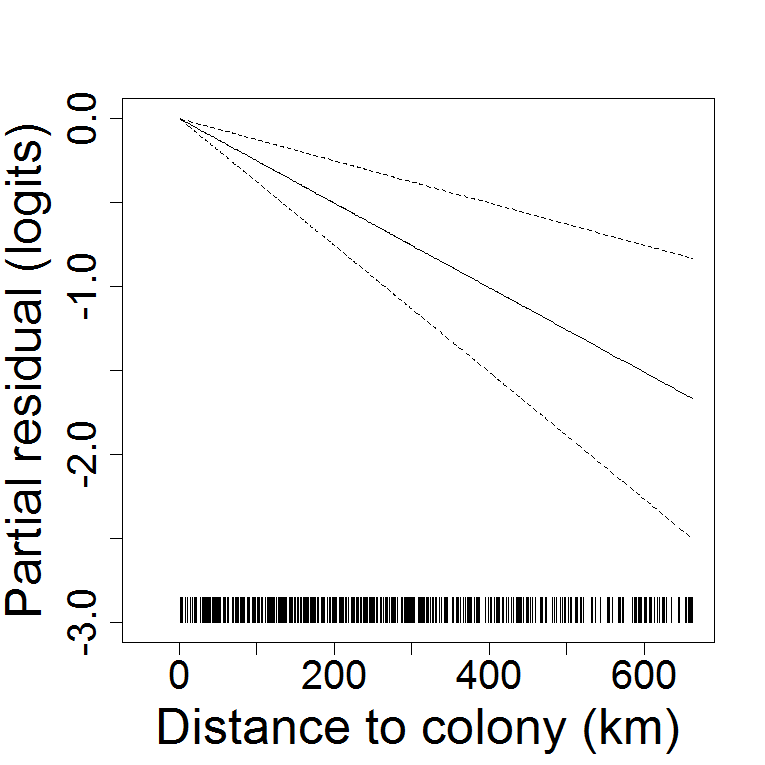

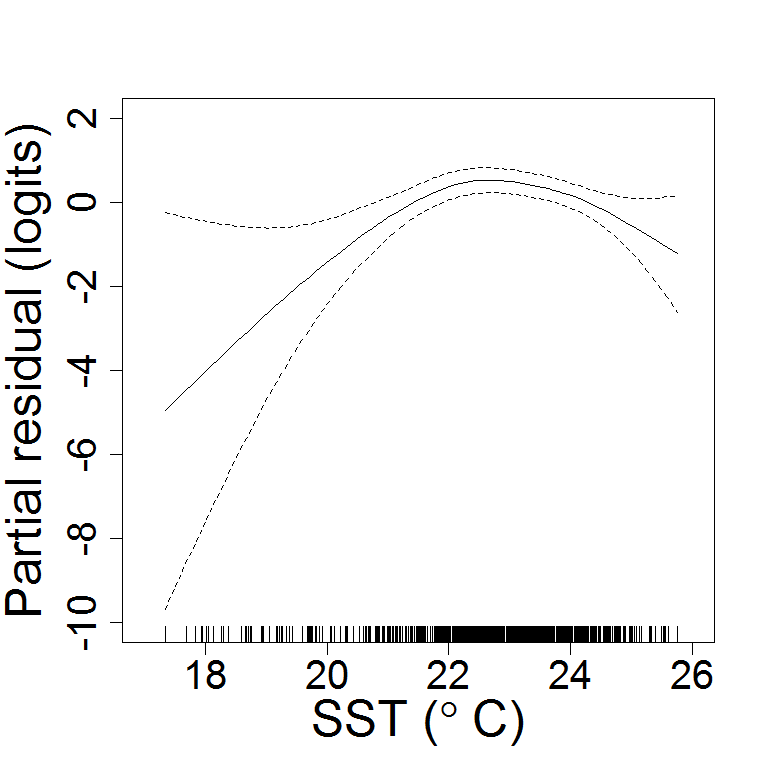


b


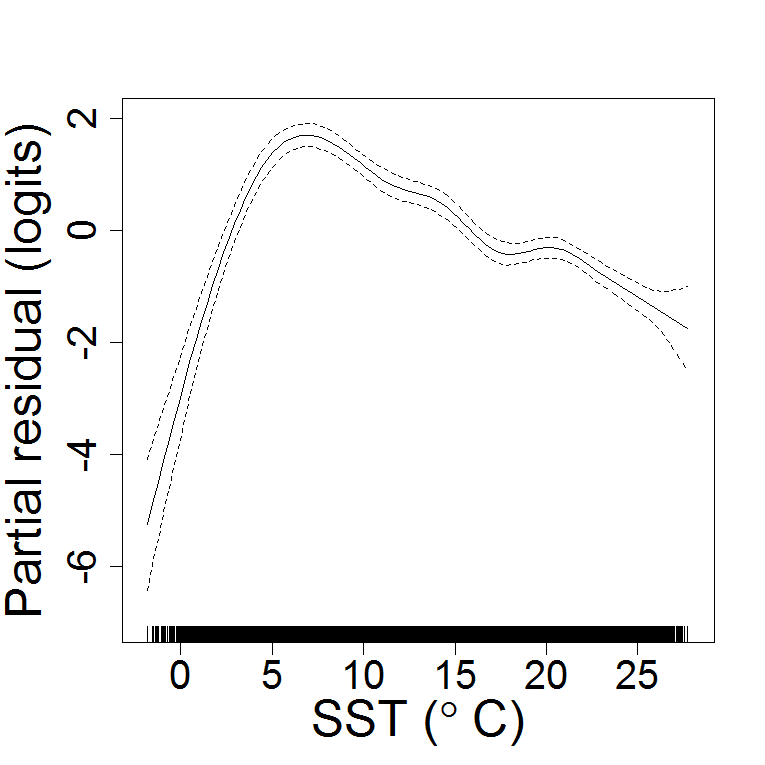

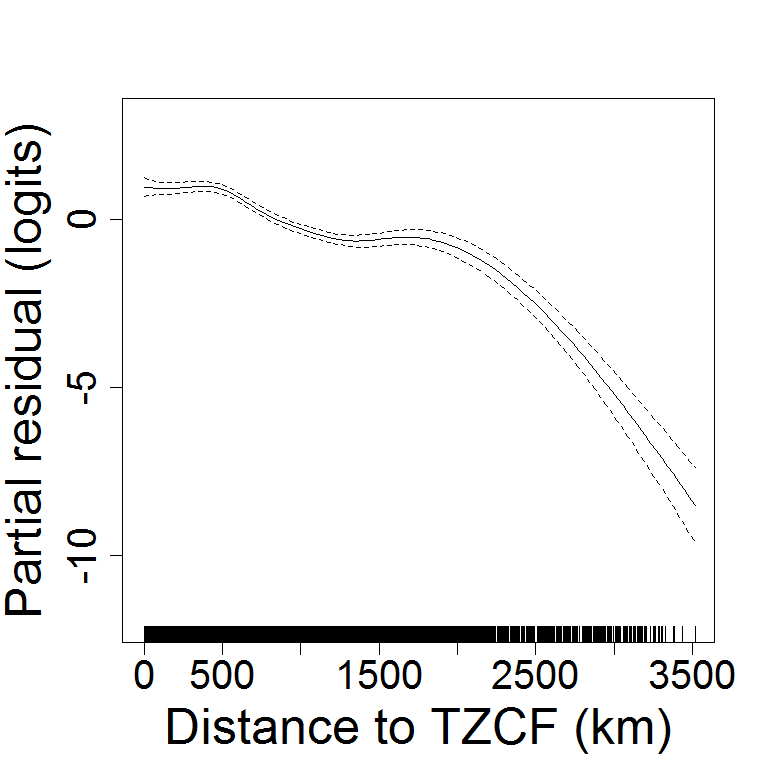

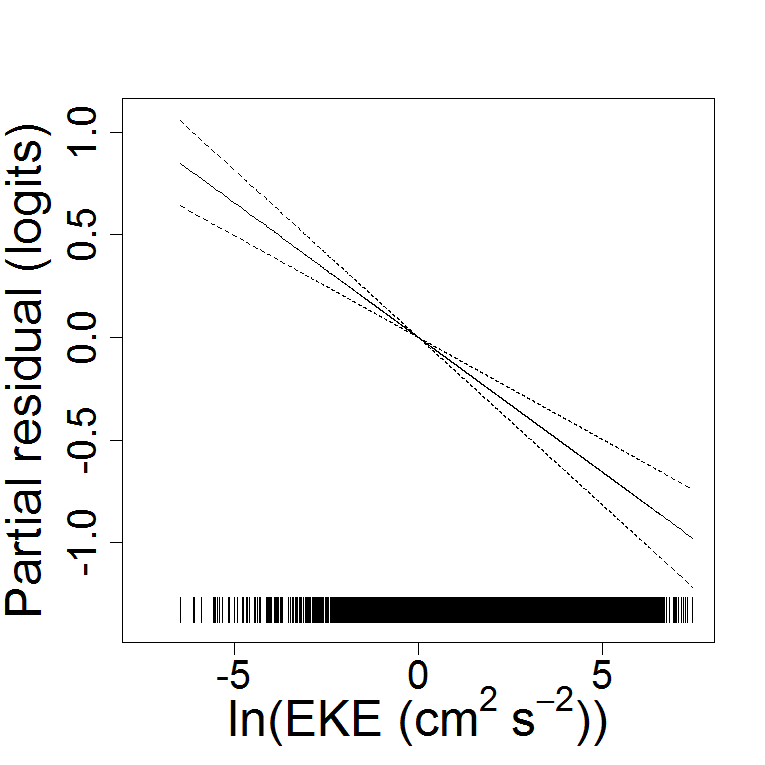


c


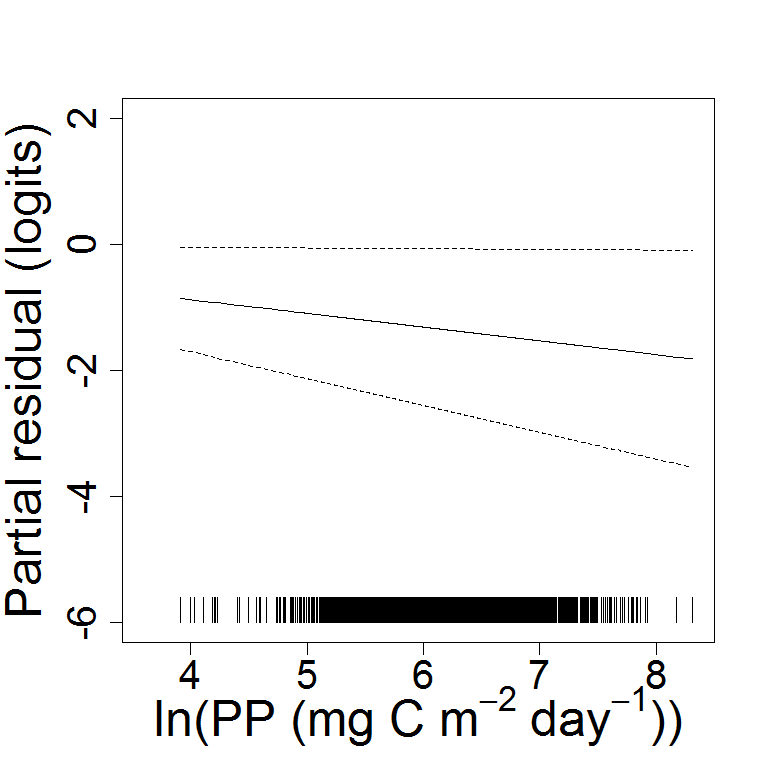

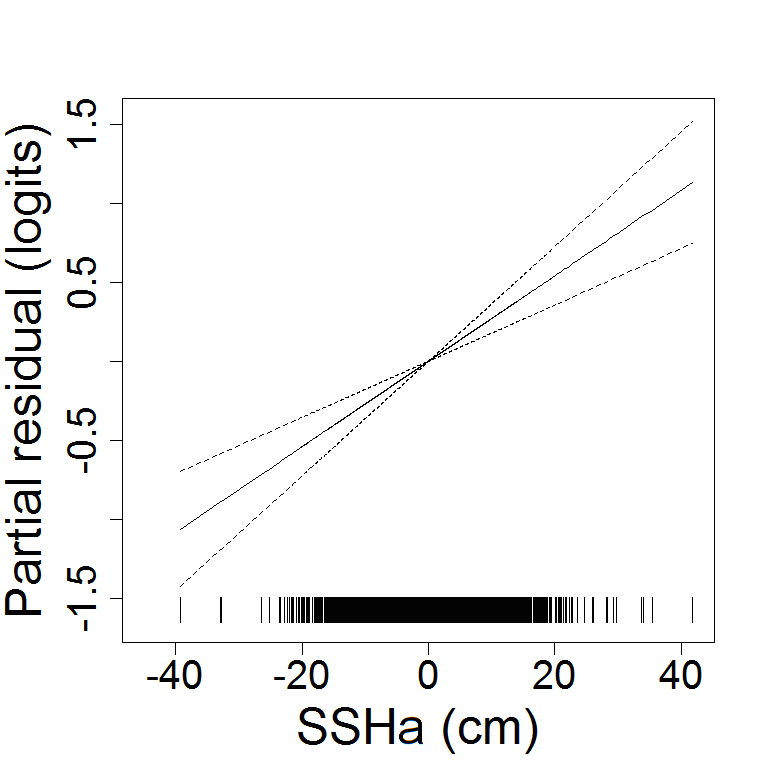

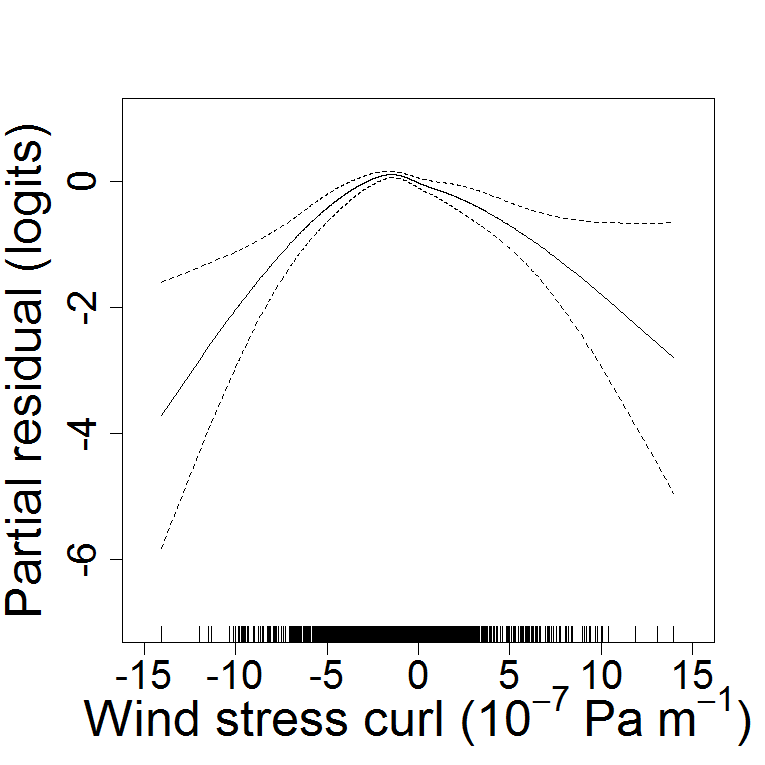


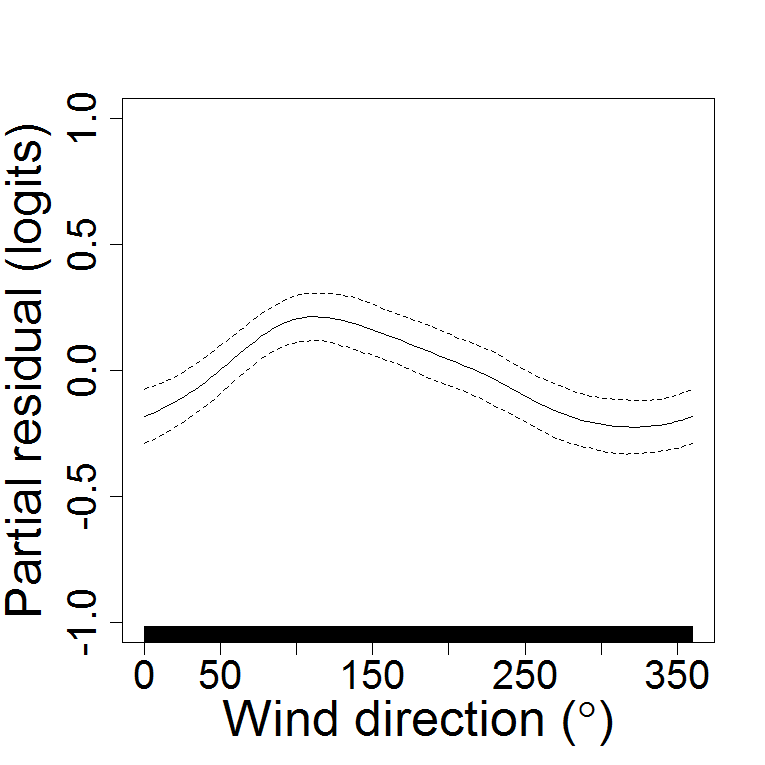

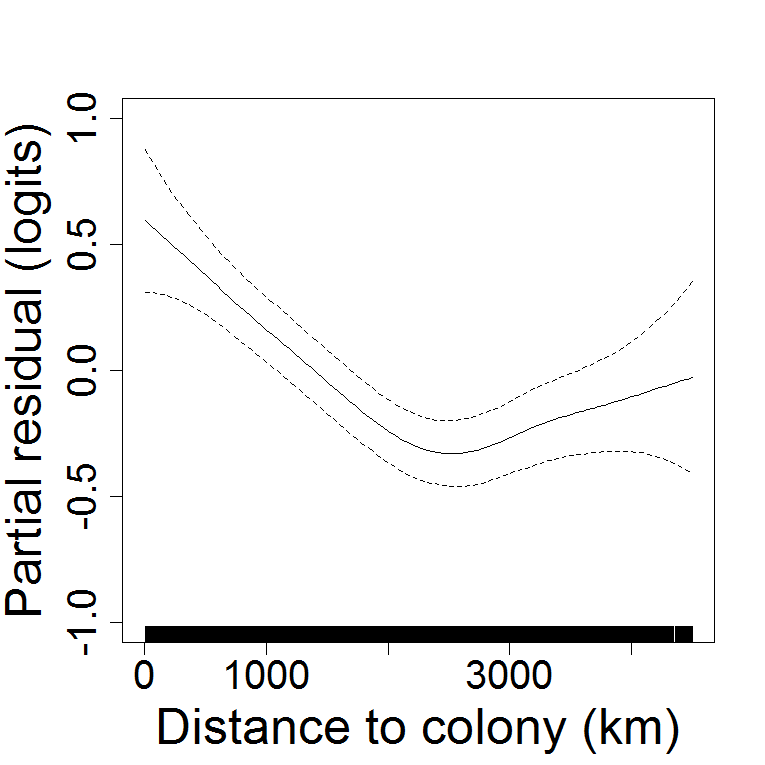

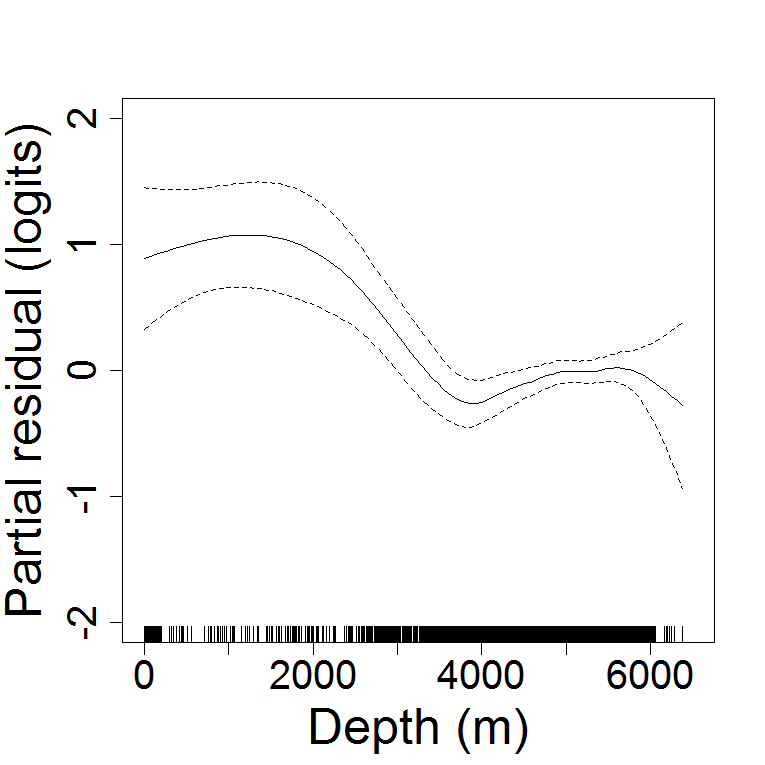


**References**

1. Wood SN. Generalized additive models: an introduction with R. Boca Raton, Florida: Chapman & Hall; 2006.

2. Aarts G, MacKenzie M, McConnell B, Fedak M, Matthiopoulos J. Estimating space-use and habitat preference from wildlife telemetry data. Ecography. 2008;31:140-160.
